# Differential responses of chili varieties grown under cadmium stress

**DOI:** 10.1186/s12870-023-04678-x

**Published:** 2024-01-02

**Authors:** Sundas Sana, Musarrat Ramzan, Samina Ejaz, Subhan Danish, Saleh H. Salmen, Mohammad Javed Ansari

**Affiliations:** 1 Department of Botany, Faculty of Chemical and Biological Sciences, The Islamia University of Bahawalpur, Bahawalpur, 63100 Pakistan; 2 Department of Biochemistry, Institute of Biochemistry, Biotechnology and Bioinformatics (IBBB), The Islamia University of Bahawalpur, Bahawalpur, 63100 Pakistan; 3 Department of Soil Science, Faculty of Agricultural Sciences and Technology, Bahauddin Zakariya University, Multan, Punjab Pakistan; 4 Department of Botany and Microbiology, College of Science, King Saud University, PO Box -2455, Riyadh, 11451 Saudi Arabia; 5 Department of Botany, Hindu College Moradabad (MJP Rohilkhand University Bareilly), Moradabad, 244001 India; 6 Al-Waili foundation of Science, New York, USA

**Keywords:** Cadmium, *Capsicum annuum* L, Flavonoids, Antioxidants, Total soluble sugars, Total proteins, Amino acids

## Abstract

Heavy metal cadmium (Cd) naturally occurs in soil and is a hazardous trace contaminant for humans, animals, and plants. The main sources of Cd pollution in soil include overuse of phosphatic fertilizers, manure, sewage sludge, and aerial deposition. That’s why an experiment was conducted to analyze the effect of Cd toxicity in *Capsicum annuum* L. by selecting its seven varieties: Hybrid, Desi, Sathra, G-916, BR-763, BG-912, and F1-9226. Cadmium was spiked in soil with four levels, i.e., (0, 3, 4, and 5 mg Cd kg^− 1^ of soil) for a week for homogeneous dispersion of heavy metal. Chili seeds were sown in compost-filled loamy soil, and 25-day-old seedlings were transplanted into Cd-spiked soil. Cadmium increasing concentration in soil decreased chili growth characteristics, total soluble sugars, total proteins, and amino acids. On the other hand, the activities of antioxidant enzymes were increased with the increasing concentration of Cd in almost all the varieties. Treatment 5 mg Cd/kg application caused − 197.39%, -138.78%, -60.77%, -17.84%, -16.34%, -11.82% and − 10.37% decrease of carotenoids level in chili V2 (Desi) followed by V4 (G-916), V1 (Hy7brid), V7 (F1-9226), V6 (BG-912), V5 (BR-763) and V3 (Sathra) as compared to their controls. The maximum flavonoids among varieties were in V5 (BR-763), followed by V6 (BG-912), V7 (F1-9226), V3 (Sathra) and V1 (Hybrid). Flavonoids content was decreased with − 37.63% (Sathra), -34.78% (Hybrid), -33.85% (G-916), -31.96% (F1-9226), -31.44% (Desi), -30.58% (BR-763), -22.88% (BG-912) as compared to their control at 5 mg Cd/kg soil stress. The maximum decrease in POD, SOD, and CAT was − 31.81%, -25.98%, -16.39% in chili variety V7 (F1-9226) at 5 mg Cd/kg stress compared to its control. At the same time, maximum APX content decrease was − 82.91%, followed by -80.16%, -65.19%, -40.31%, -30.14%, -10.34% and − 6.45% in V4 (G-916), V2 (Desi), V3 (Sathra), V6 (BG-912), V1 (Hybrid), V7 (F1-9226) and V5 (BR-763) at 5 mg Cd/kg treatment as compared to control chili plants. The highest CAT was found in 5 chili varieties except Desi and G-916. Desi and G-916 varieties. V5 (BR-763) and V6 (BG-912) were susceptible, while V1 (Hybrid), V3 (Sathra), and V7 (F1-9226) were with intermediate growth attributes against Cd stress. Our results suggest that Desi and G-916 chili varieties are Cd tolerant and can be grown on a large scale to mitigate Cd stress naturally.

**Supplementary Information:**

The online version contains supplementary material available at 10.1186/s12870-023-04678-x.

## Introduction

Heavy metals induced toxicity is one of major environmental abiotic factor which need scientific attention for achievement of sustainable crops productions [1, 2]. Heavy metals (metals with high atomic weight/number/density) and nanomaterials are released into the environment due to anthropogenic activities like industrialization and ever-increasing urbanization, which cause phytotoxicity by adversely affecting plant physiology and development [3]. All heavy metals are non-biodegradable and cannot be naturally removed from the environment by any conceivable natural means. Some heavy metals are mobile (can be taken up by plant roots via diffusion, endocytosis, or through metal transporters), and few are immobile (cannot move from the place where they are accumulated) [4]. In addition to the direct effects of bioactive metals on plants, excessive production of reactive oxygen species (ROS) can also cause oxidative stress and cell damage [5].

Heavy metal Cd is ranked the 7th most dangerous material on the 2017 Hazardous Substances Priority List by the Agency for the Registration of Dangerous Substances and Diseases [6]. Cadmium lacks a specialized plant transporter; it often enters plant tissues by competing with divalent metal ion transporters like those for zinc, iron, and manganese [7]. Reactive oxygen species (ROS) are accumulated in significant quantities by plants under Cd stress, which increases membrane fluidity and permeability and disturbs the plasma membrane system [8].

Cadmium interferes with chlorophyll biosynthesis, activates or inhibits several Calvin cycle enzymes, deteriorates the evolution of oxygen over photosystem II (PSII), interferes with the transfer of electrons between PSI and PSII, and inhibits the activity of several enzymes, including carbonic anhydrase, and phosphoenolpyruvate carboxy [9]. As a result, Cd poisoning can cause a variety of alterations in plant bodies, including morphological, physiological, biochemical, and photochemical changes [10]. Due to its high-water solubility and ease of uptake, Cd is a highly phytotoxic non-essential element for plants [11, 12]. The activities of many enzymes, including those involved in photosynthesis, CO_2_ fixation, carbohydrate metabolism, and Rubisco, could also be significantly altered by Cd [13]. Additionally, Cd has been shown to interfere with cell signaling and expression of genes [14].

To mitigate the hazardous effects of abiotic stresses, superoxide dismutase (SOD), catalase (CAT), ascorbate peroxidase (APX), glutathione reductase (GR), and dehydroascorbate reductase (DHAR) are some of the antioxidative enzymes and non-enzymatic antioxidants that plants use to scavenge ROS [15]. The ability of plants to withstand abiotic stress may also be increased by non-enzymatic antioxidants, which may directly detoxify oxygen free radicals in plants [16]. According to Faiz et al., [17] and Taie et al., [18], plant tolerance mechanisms include a variety of intricate processes, including adjustments in proteins, gene expression, different metabolites (primary and secondary), and the development and activation of antioxidant machinery to scavenge oxidative stress markers.

Chili pepper, *Capsicum annuum* L., is grown all over the world. Chili productivity for spices and vegetables has expanded significantly over the years. Egypt was listed among the top 20 *C. annuum*-producing countries, with a planted area of roughly 17,306 hectares and a dry fruit production of approximately 60,194 tons [19]. It includes capsaicin and is high in provitamins A, C, and E, minerals, antioxidants, and secondary compounds (carotenoids, phenolic acids, flavonoids, and alkaloids) [20]. Dry fruits have traditionally been used as spices, but they are also employed in industrial processes, pharmaceuticals, and cosmetics [21].

As different plant species exhibit genetic variation the selection of a variety that can withstand under Cd stress may have a potential for improving chili yield at contaminated site. The current study utilized phenotypic characteristics to describe the Cd tolerance of *Capsicum annuum* L. genotypes. The study aimed to select chili varieties tolerant to Cd stress on the basis of morphological attributes and antioxidant activities.

## Materials and methods

### Experimental site and treatments

A pot experiment was performed in the Department of Botany, Islamia University of Bahawalpur, Pakistan (the latitude of Bahawalpur, Pakistan, is 29.39, and the longitude is 71.68; Bahawalpur is located with the GPS coordinates of 29° 24’ N and 71° 40’ E. The geographical domain of the study area was the district Bahawalpur, which is situated in southern Punjab, Pakistan. The study area is approximately 50 km from east to west and 47 km from north to south, which covers 2372 km^2^. The temperature ranges between 24.5 and 52 °C in summer and in winter ranges between 10.9 and 20.3 °C. District Bahawalpur falls under a semi-arid region with the cultivation of cotton and wheat crops. A total of 7 varieties of *Capsicum annuum* L. were obtained from the SKY Seed Store, Lahore, Punjab, Pakistan. Seeds were first placed in compost and loamy soil-filled pots and incubated at 25 °C for successful germination. Pots were watered regularly to ensure the moisture content.

### Soil spiking with cadmium

Loam texture soil was collected from the nursery of Islamia University, Bahawalpur, and used for the growth of chili varieties. Five kilogram of soil was added to each plastic bag and spiked with Cd levels (0, 3, 4, 5 mg Cd/kg soil). Cadmium-spiked soil was mixed thoroughly for one week to disperse the heavy metal. Twenty-five days old chili seedlings (3 seedlings per bag) were transplanted into the spiked soil of plastic bags (27.9 × 17.78 cm). The experimental set-up contains control (untreated) and three Cd treatments (with three replicates of each treatment). Chili seedlings were watered regularly with 50 ml of distilled water for 15 days after Cd treatment exposure. After 15 days of Cd treatment exposure, chili seedlings were harvested, washed, and collected in plastic zipper bags for laboratory morphological, physiological, and biochemical analysis.

### Chemical analysis of the soil

Soil analyses were also performed before soil spiking with Cd (Table 1). Loamy soil was used for this research work. Soil samples were sieved through a 2 mm screen and dried at a natural air temperature. pH value was determined by using saturated soil paste extract. A conductivity meter was used to measure the electrical conductivity of soil saturation paste extract. Soil texture was also determined. The soil’s micronutrient content (potassium and phosphorus) was analyzed by [22]. Heavy metals (Mn, Zn, Cu, Fe) were analyzed in the digested samples by using atomic absorption spectrophotometer [23]. Boron was measured by following the method of [24].

**Table 1:** Soil properties for pot experiment

Soil Property	Unit	Parameter Value	Reference
Soil Depth	cm	0–15	[25]
Texture	-	Loam	
Zn	(mg/kg)	2.78	[26]
Cu	(mg/kg)	1.23	
Fe	(mg/kg)	3.20	
Mn	(mg/kg)	0.56	
B	(mg/kg)	0.44	[24]
EC	(mS cm^− 1^)	2.3	[27]
pH	-	8.16	[28]
Organic Matter	(%)	0.84	[29]
P	(mg kg^− 1^)	4.09	[22]
K	(mg kg^− 1^)	138	
Saturation	(%)	38	[30]

### Cadmium accumulation in root and shoot

Cadmium concentration (mg/kg DW) in plant roots and shoots was determined using chili plant seedlings. The Chapman and Pratt technique used atomic Absorption (Perkin-Elmer, Model 330) [31]. The shoot and root samples (0.1 g each) were dried and powdered before being digested for 12 h with nitric acid and perchloric acid. Before determining the Cd^2+^ level, all digested samples were diluted in 100 ml of distilled water.

### Morphological analysis

Shoot length (SL), root length (RL), number of leaves, number of roots, and leaf area (LA) of all treated and control chili plants were measured. Plant sample’s fresh and dry weight was measured separately using an electronic balance. One plant of each treatment was dried by oven drying method at a constant temperature of 100–105 °C for 4 h., and dry weight was measured.

### Translocation factor and metal tolerance index

The metal translocation factor was calculated by using the following formula of Adesodun et al., [32].

$${\rm{Translocation}}\,{\rm{Factor}}\left( {{\rm{TF}}} \right) = \left( {\frac{{{\rm{Cshoot}}}}{{{\rm{Croot}}}}} \right) \times 100$$

C_shoot_, is the concentration of heavy metal in the shoot and C_root_ is the concentration of heavy metal in the root.

The heavy metal tolerance index was calculated by [33] following the formula:

$$\begin{array}{l}{\rm{Metal}}\,{\rm{Tolerance}}\,{\rm{Index}}\left( {{\rm{MTI}}} \right) = \\\,\,\,\,\,\,\,\,\,\,\,\,\,\,\,\,\,\,\,\,\,\left( {\frac{{{\rm{Growth}}\,{\rm{parameter}}\,{\rm{in}}\,{\rm{contaminated}}\,{\rm{soil}}}}{{{\rm{Growth}}\,{\rm{parameter}}\,{\rm{in}}\,{\rm{control}}\,{\rm{soil}}}}} \right) \times 100\end{array}$$

### Chlorophyll contents

To estimate the chlorophyll in leaves, Brougham’s method was employed. One gram’s sample of green leaf was weighed and ground in a mortar and pestle that had been allowed to cool. The chlorophyll content was eliminated through repeated homogenization using 80% cooled acetone (20 mL distilled water + 80 mL acetone). The supernatant was filtered before being diluted in 80% acetone to 100 ml [34]. Using Arnon [35] method, which involved measuring absorbance in a double-beam UV spectrophotometer (Systronics 128) at 663, 645, 537, and 480 nm, the quantity of chlorophyll a and b was determined.

$${\rm{Chlorophyll}}\,{\rm{a}}\left( {\frac{{{\rm{mg}}}}{{\rm{g}}}} \right) = \frac{{\left( {12.7 \times {\rm{A}}663} \right) - \left( {2.69 \times {\rm{A}}645} \right) \times {\rm{V}}}}{{1000 \times {\rm{W}}}}$$

$${\rm{Chlorophyll}}\,{\rm{b}}\left( {\frac{{{\rm{mg}}}}{{\rm{g}}}} \right) = \frac{{\left( {22.9 \times {\rm{A}}645} \right) - \left( {4.68 \times {\rm{A}}645} \right) \times {\rm{V}}}}{{1000 \times {\rm{W}}}}$$

$${\rm{Total}}\,{\rm{Chlorophyll}}\left( {\frac{{{\rm{mg}}}}{{\rm{g}}}} \right) = \frac{{20.2\left( {{\rm{OD}}645} \right) + 8.02\left( {{\rm{OD}}663} \right) \times {\rm{V}}}}{{1000 \times {\rm{W}}}}$$

$$\begin{array}{l}{\rm{Carotenoids}}\left( {\frac{{{\rm{mg}}}}{{\rm{g}}}} \right) = {\rm{OD}}480 + 0.114\left( {{\rm{OD}}663} \right)\\\,\,\,\,\,\,\,\,\,\,\, - 0.638\left( {{\rm{OD}}645} \right)\end{array}$$

$$\begin{array}{l}{\rm{Anthocyanin}}\left( {\frac{{\mu {\rm{mol}}}}{{{\rm{ml}}}}} \right) = 0.08173\left( {{\rm{OD}}537} \right)\\- 0.00697\left( {{\rm{OD}}645} \right) - 0.002228\left( {{\rm{OD}}663} \right)\end{array}$$

Where,

A/OD = Absorbance of chlorophyll extract on specific induced wavelength.

V = Final volume of extract in a mixture of 80% acetone.

FW = Fresh weight of tissue (mg).

### Total protein estimation

‘Bradford Protein Assay’ was used to measure the concentration of total proteins in a solution. The main principle of this assay is the binding of sample amino acid proteins with Coomassie Brilliant Blue G-250, which changes color from brown to blue when absorption was taken at 595nmsing a spectrophotometer [36].

### Total soluble sugar and flavonoid content

The anthrone method described by Chow & Landhäusser [37] was used to calculate total soluble sugar after making minor changes. The supernatant was combined with 750 μl of anthrone reagent in a heat block (Eppendorf Thermomixer Compact 5,350 Mixer) and heated at 100 °C for 10 min. The reaction tubes were submerged in ice for ten minutes. A blank tube containing 50 μl of 80% ethanol was added to 750 μl of anthrone reagent and incubated under the same conditions. After 150 μl of sample, standard, or blank were transferred from the assay tube to a see-through 96-well microplate, the absorbance of each well was measured at 625 nm. The TSS content was determined using a D-glucose standard curve in 80% ethanol (1 to 0 mg/ml). Each absorbance measurement was performed three times.

At 25 °C, fresh leaf material (500 mg) was crushed and extracted in ethyl alcohol. The colorimetric method of Zhishen et al. [37] measured flavonoid content. Catechin extract was employed as a reference for the calibration curve. A spectrophotometer (Beckman 640D, USA) measured absorbance at 510 nm. Flavonoid concentration was measured in mg catechin equivalents per gram of extract.

### Total amino acids

The manufacturer’s instructions for the commercial ninhydrin reagent were followed with a few minor modifications when determining the free amino acids. 150 μl of supernatant and 75 μl of ninhydrin reagent solution were mixed in a microcentrifuge tube. The tubes were heated to 100 °C in a heat block for 10 min. The blank tube contained 150 μl of 80% ethanol and 75 μl of ninhydrin reagent solution. Each reaction tube received 375 μl of 95% ethanol after being chilled on ice. After 150 μl of sample, standard, or blank were transferred from the assay tube to a transparent 96-well microplate, the absorbance of each well was measured at 440, 520, and 570 nm [38]. FAA content was reported as mg of an equal amount of L-proline and L-glycine equivalents per ml using a calibration curve made with standard solutions of L-proline and L-glycine (1 to 0 mg/ml). Three measurements of absorbance were made for each. The ninhydrin reagent was prepared using the Moore et al. [39] method.

### Determination of H_2_O_2_ content and MDA

The H_2_O_2_ test is based on the oxidation of ferrous ions in the presence of the ferric ion indicator xylenol orange [40]. Cavalcanti et al. [41] described a method for measuring lipid peroxidation in terms of malondialdehyde (MDA) content. At 4 °C, samples (0.5 g) were homogenized in 4 ml of 1% (w/v) trichloroacetic acid (TCA) with a mortar and pestle. Homogenates were centrifuged for 20 min at 12,000 g. A reaction mixture (3 ml) containing 20% (w/v) TCA and 0.5% (w/v) thiobarbituric acid (TBA) was added supernatant (1 ml). The mixture was incubated at 95 °C for 30 min before being swiftly placed in a cold bath to cease the process. The fraction’s absorbance was measured at 440, 532, and 600 nm.

### Temporal study of antioxidant enzymes

The antioxidant enzyme assays (peroxidases, superoxide dismutases, catalases, and ascorbate peroxidases) were measured by following their respective protocols. Distilled water inoculated samples will be used as a control group. One milliliter of 50 mM buffer (potassium-phosphate) with a pH of 7 was used to homogenize one gram of chili seedling. The resulting mixture was centrifuged for 15 min at 4 °C using a 12,000-rpm motor.

#### Peroxidase assay

Peroxidase was evaluated using the Chance & Maehly [42] approach. The peroxidation with an electron donor (guaiacol) and the production of tetra guaiacol were used to determine activity. In 0.1 ml of sample enzyme extract, 50 mM potassium phosphate buffer pH 5, 20 mM guaiacol, and 40 mM H_2_O_2_ were mixed. Optical density was calculated at 470 nm every 20 s.

#### Catalase assay

The approach given by Chance & Maehly [42] was used to analyze CAT activity. The reaction mixture (3 mL) was tested, which contained 50 mM phosphate buffer (pH 0.0), 5.9 mM H_2_O_2_, and 0.1 mL enzyme extract. The CAT activity was determined by measuring the change in absorbance owing to H_2_O_2_ intake at 240 nm every 20 s.

#### Superoxide dismutase assay

The ability of SOD to prevent the photochemical reduction of nitro blue tetrazolium (NBT) [43]. At 560 nm, the optical density (OD) was measured. The reaction mixture comprises 50 mM sodium phosphate buffer (pH 7.8), 13 mM methionine, 2 mM riboflavin, 75 mM NBT, 100 mM EDTA, and 100 mL enzyme extract.

#### Ascorbate peroxidase assay

By evaluating the decrease in absorbance at 290 nm, APX activity was identified [44]. The 50 mM phosphate buffer (pH 7.6), 0.1 mM sodium-EDTA, 12 mM H_2_O_2_, 0.25 mM ascorbic acid, and plant extract are all combined in 1 ml of reaction volume.

### Statistical analysis

The mean of three replicates was used to represent all experiment data. Origin 2021 Pro software was used for making graphs [45]. A paired comparison using two-way factorial analysis was executed. The treatment means were compared by using Fisher’s LSD test with *p* < 0.05 used as a significant level.

## Results

### Effect of cd on growth parameters of Chili

Treatment impacts were significant on morphological attributes, i.e., shoot length, root length, plant fresh weight, plant dry weight, leaf area, number of leaves, and number of roots under different levels of Cd in Capsicum varieties. Soil application of 3, 4 and 5 mg Cd/kg soil significantly decreased the growth parameters of chili as compared to control seedlings in all seven varieties. SL, RL, PFW and PDW, LA were maximum in Variety 4 (G-916) and Variety 2 (Desi) at higher levels of Cd treatment and were tolerant to Cd stress levels. V5 (BR-763) and V6 (BG-912) have the least shoot length, root length, plant fresh and dry weight and were susceptible to Cd treatments. Capsicum V1 (Hybrid), 3 (Sathra) and 7 (F1-9226) had intermediate growth attributes against Cd stress. *Capsicum annuum* L. plant height and weight were decreased significantly by 3, 4 and 5 mg Cd/kg soil in all varieties, respectively. More reduction in these parameters was at a higher stress level of 5 mg Cd/kg soil in all seven *Capsicum annuum* L. varieties.

**Fig. 1:**
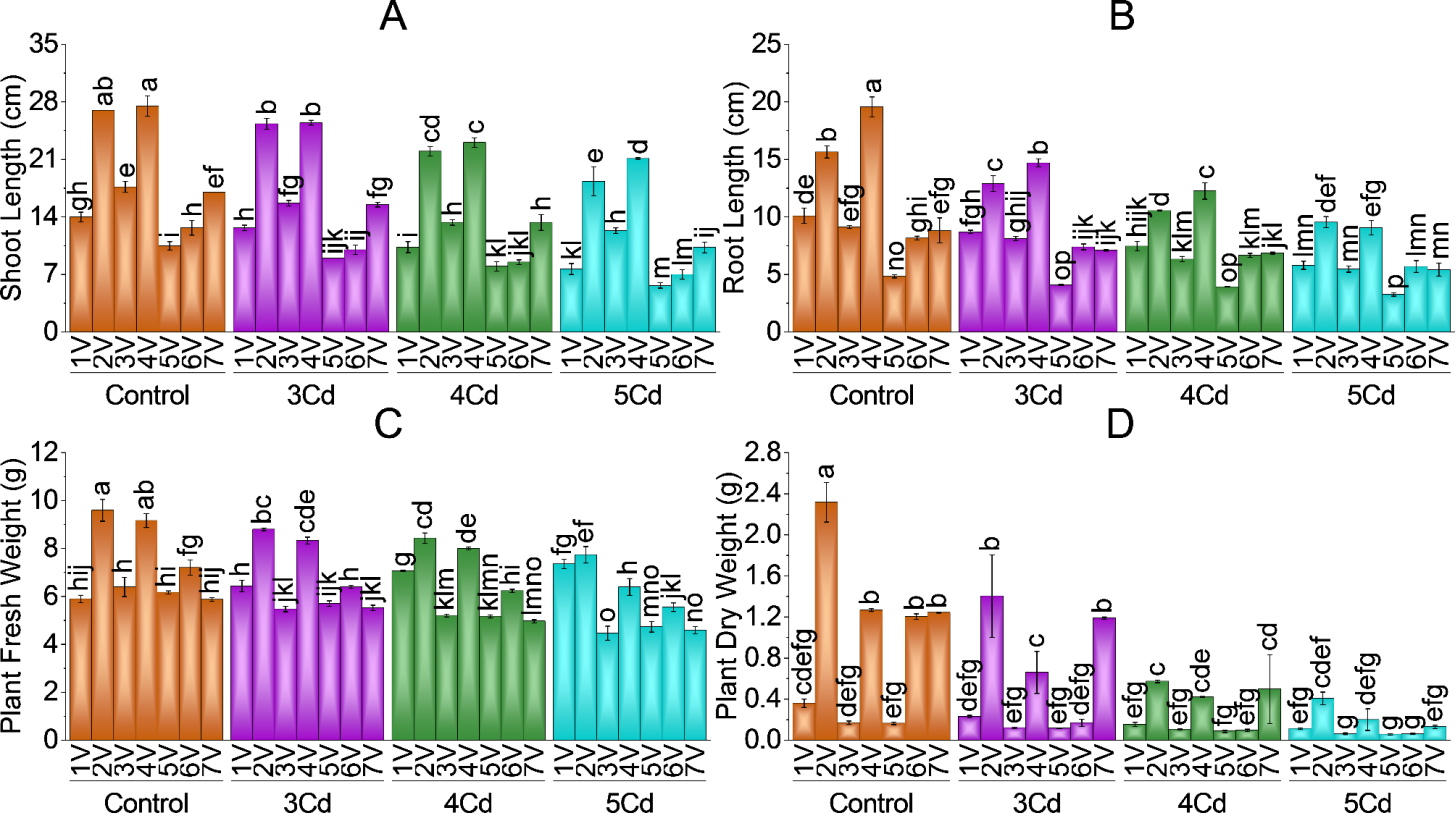
Effect of variable toxicity levels of Cd on shoot length (**A**), root length (**B**), plant fresh weight (**C**) and plant dry weight (**D**) of different chili varieties. Bars are means ± SE of 3 replicates. Different bar letters showed significant changes at *p* ≤ 0.05; Fisher’s LSD

The greatest Cd concentration (5 mg Cd/kg soil) caused the most damage. It decreased shoot length by 46.03% in V5-BR-763 followed by V1-Hybrid (45.24%) V6-BG-912 (44.74%), V7-F1-9226 (39.41%), V2-Desi (32.10%), V3-Sathra (30.19%) and V4-G-916 (23.27%) respectively as compared to their controls (Fig. 1-A). At 3 and 4 mg Cd/kg soil treatment, a maximum decrease in shoot length was observed in V6 (BG-912) with 21.05% and 32.89%, followed by V1 (Hybrid) with 9.48% and 26.19%, respectively. Root length was decreased by (53.66%) V4-G-916, (42.57%) V1-Hybrid, (40.15%) V3-Sathra, 38.49% (V7-F1-9226), (38.98%) V2-Desi, 32.41% (V5-BR-763) and (30.02%) V6-BG-912 at highest level of Cd stress application compared to their controls (Fig. 1-B). Maximum reduction in root length at 3 and 4 mg Cd/kg soil treatment was observed as 24.86% and 37.31% in V4 (G-916), followed by V3 (10.95% and 30.66%), V1 (13.86% and 26.07%) respectively as compared to their control group.

Plant fresh weight was with decreasing trend in V4 (G-916), V3 (Sathra), V5 (BR-763), V6 (BG-912), V7 (F1-9226) and V2 (Desi) with 30.22%, 30.21%, 23.24%, 22.92%, 21.74% and 19.32% at 5 mg Cd/kg soil treatment as compared to their controls (Fig. 1-C). In V1 (Hybrid), plant fresh weight was increased by -9.04%, -19.77% and − 24% at 3, 4 and 5 mg Cd/kg soil application compared to the control. Plant dry weight was with decreasing trend of 94.74%, 89.54%, 84.21%, 82.45%, 68.52%, 65.31% and 62.75% V6, V7 and V4, V2, V1, V5 and V3 as compared to their control group at 5 mg Cd/kg soil treatment (Fig. 1-D).

Applying Cd stress significantly decreased leaf area, number of leaves and roots in BR-763, BG-912 and F1-9226 capsicum varieties. Desi and G-916 were tolerant to 5 mg Cd/kg level stress with better growth. The control group showed maximum LA, no leaves, and number of roots compared to treated ones. Moderated growth was recorded at medium levels of Cd (3 and 4 mg Cd/kg soil) in all chili varieties.

**Fig. 2:**
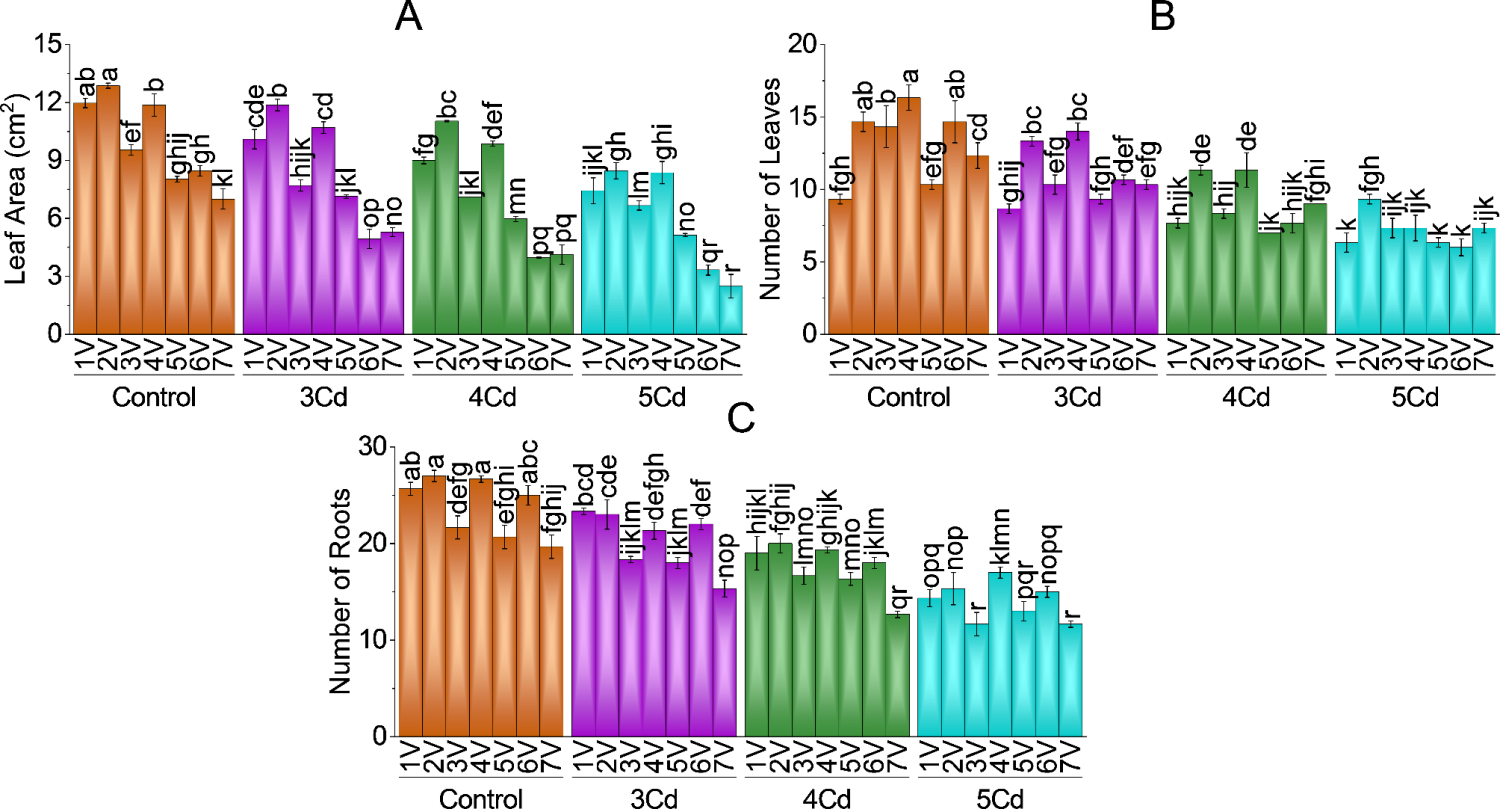
Effect of variable toxicity levels of Cd on leaf area (**A**), number of leaves (**B**) and number of roots (**C**) of different chili varieties. Bars are means ± SE of 3 replicates. Different bar letters showed significant changes at *p* ≤ 0.05; Fisher’s LSD

Reduction in chili leaf area was 64.47%, 60.67%, 37.88%, 36.10%, 34.20%, 30.17%, 29.49% in F1-9226 (V7), BG-912 (V6), Hybrid (V1), BR-763 (V5), Desi (V2), Sathra (V3) and G-916 (V4) as compared to their controls as 5 mg Cd/kg soil stress. Leaf area was reduced by 41.69% and 53.15% in V6 (BG-912) and 24.52% and 40.49% in V7 (F19226) at 3 and 4 mg Cd/kg soil stress (Fig. 2-A). Cd application (5 g/kg) reduced the number of leaves by 59.09%, 55.10%, 48.84%, 40.67%, 38.71%, 36.36%, and 32.14% in V6 (BG-912), V4 (G-916), V3 (Sathra), V7 (F1-9226), V5 (BR-763), V2 (Desi) and V1 (Hybrid) as compared to their controls. At 3 and 4 mg Cd/kg soil application, reduction in the number of leaves was more in V6 (BG-912) V3 (Sathra), V4 (G-916) with (27.27%, 47.73%), (27.91%, 41.86%) and (14.29%, 30.61%,) respectively as compared to control plants (Fig. 2-B). Number of roots was least in V3 (Sathra), V7 (F1-9226) and V6 (BG-912) with (15.38%, 23% and 46.84%), (22.03%, 35.59% and 40.67%) and (12%, 28%, 40%) at 3, 4 and 5 mg Cd/kg soil application respectively, as compared to its control (Fig. 2-C).

### Photosynthetic and accessory pigments

The photosynthetic (chlorophyll a, chlorophyll b, and total chlorophyll) and accessory pigment (carotenoids) were significantly (*p* *≤* 0.05) affected by Cd treatment. Chlorophylls a, b, and total chlorophylls and carotenoids were comparatively low in Cd-treated varieties. The application of Cd highly influenced these pigments of *Capsicum annuum* L. varieties with a maximum in control and decreased trend in 3, 4 and 5 mg Cd/kg. Chl a, Chl b and Tot. Chl was maximum in V2 and V4 (Desi and G-916) at 3 and 4 mg Cd/kg soil stress. At 5 mg Cd/kg soil application, photosynthetic pigments decreased significantly compared to the control.

**Fig. 3:**
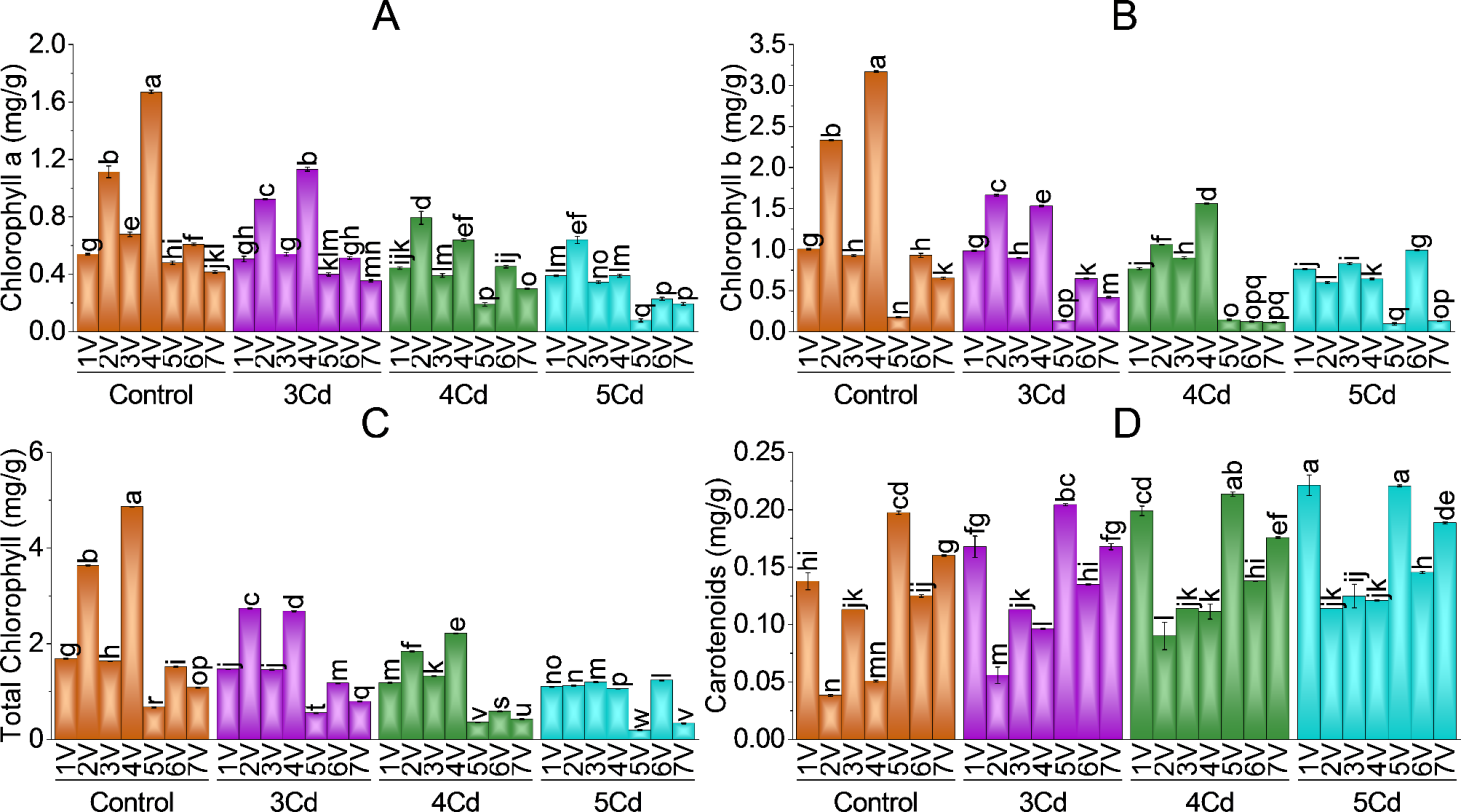
Effect of variable toxicity levels of Cd on chlorophyll a (**A**), chlorophyll b (**B**), total chlorophyll (**C**) and carotenoids (**D**) of different Capsicum varieties. Bars are means ± SE of 3 replicates. Different bar letters showed significant changes at *p* ≤ 0.05; Fisher’s LSD

Chlorophyll a and chlorophyll b and total chlorophylls were least in (V5 and V7) BR-763 and F1-9226 at 5 mg Cd/kg soil treatment. Hybrid, Sathra and BG-912 showed moderate levels of photosynthetic pigments. Chl a, was with a maximum decrease at 5 mg Cd/kg soil application in V5 (BR-763), followed by V4 (G-916), V6 (BG-912), V7 (F1-9226), V3 (Sathra), V2 (Desi), V1 (Hybrid) with 83.65%, 76.68%, 62.32%, 53.44%, 49.26%, 42.51% and 27.78% respectively as compared to their control (Fig. 3-A). Chl b was damaged more by the highest level of Cd application in V7 (F1-9226), V4 (G-916), V2 (Desi), V5 (BR-763), V1 (Hybrid) and V3 (Sathra) with 79.98%, 79.70%, 74.34%, 45.43%, 23.99% and 10.53% respectively as compared their non treated plants. Chlorophyll b was increased in V6 (BG-912) with − 6.62% at 5 mg Cd/kg soil application (Fig. 3-B). Total chlorophyll was maximumly reduced in V4 (G-916) followed by V5 (BR-763), V2 (Desi), V7 (F1-9226), V1 (Hybrid), V3 (Sathra) and V6 (BG-912) at 5 mg Cd/kg soil treatment with 78.21%, 71.44%, 69.10%, 69.08%, 35.01%, 26.78% and 18.70% (Fig. 3-C).

Carotenoids were significantly affected by Cd treatments. Accessory pigment was increased in all Capsicum varieties compared to controls (Fig. 3-D). V1, V5 and V7 (Hybrid, BR-763 and F1-9226) have maximum carotenoid content compared to other chili varieties. Variety 2 and 4 (Desi and G-916) has the least carotenoids. In contrast, V3 and V6 (Sathra and BG-912) have moderate carotenoid levels at all levels of Cd treatments. Treatment 5 mg Cd/kg soil application caused − 197.39%, -138.78%, -60.77%, -17.84%, -16.34%, -11.82% and − 10.37% decrease in chili V2 (Desi) followed by V4 (G-916), V1 (Hy7brid), V7 (F1-9226), V6 (BG-912), V5 (BR-763) and V3 as compared to their controls. Other levels of Cd (3 and 4 mg Cd/kg) also caused an increase in carotenoids content with respect to their controls.

### Total proteins, total soluble sugar, flavonoids and total amino acids

**Fig. 4:**
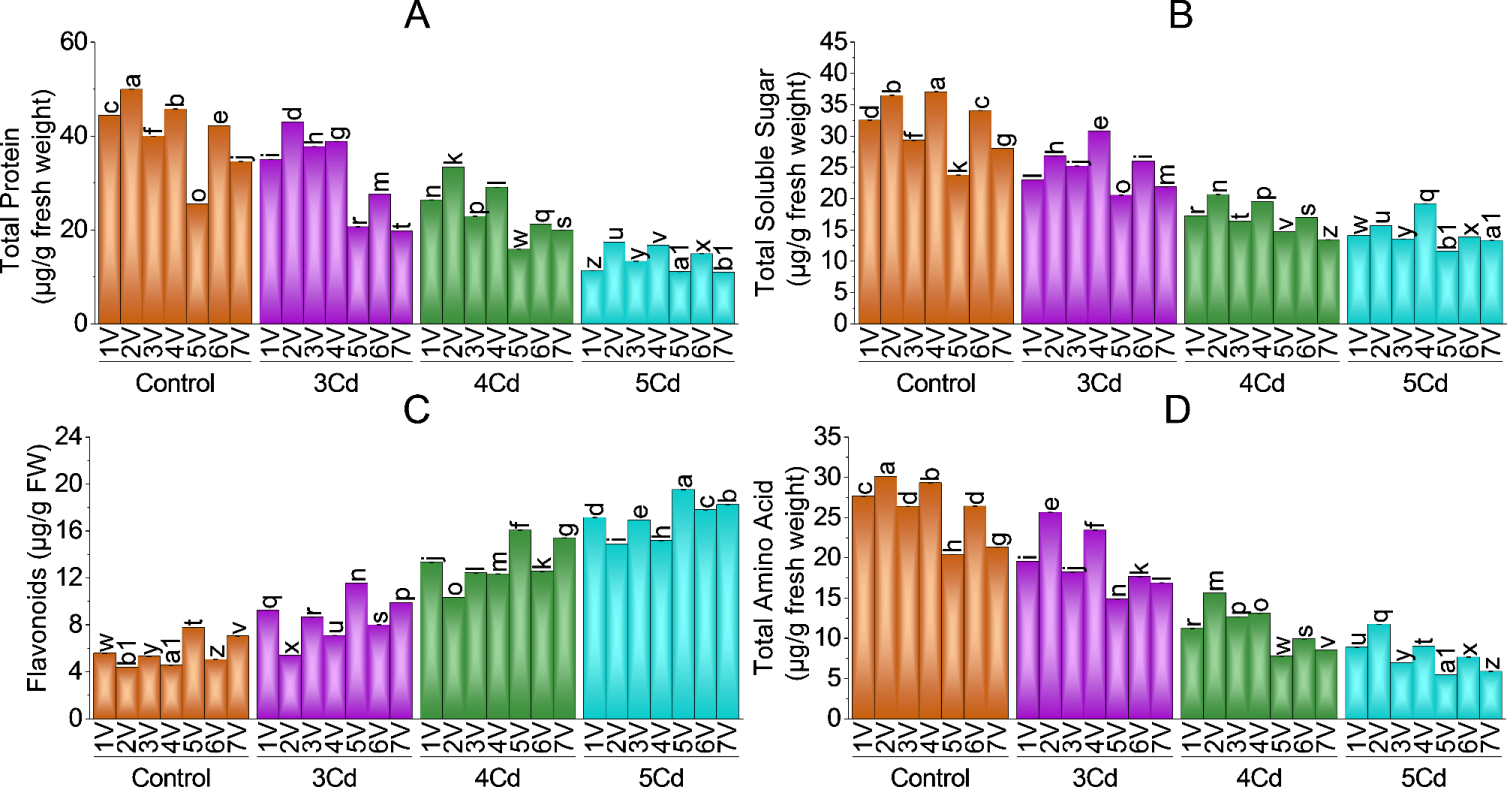
Effect of variable toxicity levels of Cd on total protein (**A**), total soluble sugar (**B**), flavonoids (**C**), and total amino acids (**D**) of different *Capsicum annuum* L. varieties. Bars are means ± SE of 3 replicates. Different bar letters showed significant changes at *p* ≤ 0.05; Fisher’s LSD

Different application rates of Cd stress significantly affected total proteins (Fig. 4-A), total soluble sugars (Fig. 4-B), flavonoid content (Fig. 4-C), and total amino acids (Fig. 4-D) of chili varieties. Total proteins and amino acids were highest in V2 and V4 (Desi and G-916) at all levels of Cd treatment. Moderate and highest Cd concentration levels (4 and 5 mg Cd/kg) decreased the total proteins and amino acids in V5 and V3 (BR-763 and Sathra). Varieties V7 and V1 (F1-9226 and hybrid) have moderate activation of total proteins and total amino acid contents. The maximum decrease in total proteins was 73.64%, 73.30% V3 (Sathra), and V5 (BR-763) compared to their controls at 5 mg Cd/kg stress. Total amino acids were increased compared to their control at all levels of Cd application. The maximum decrease was in V4 (-245.98%), V3 (-233.48%) compared to their control at 5 mg Cd/kg soil treatment.

Total soluble sugars were maximum in V4 (G-916) and in V2 (Desi) and least in V5 (BR-763) at 5 mg Cd/kg treatment among all chili varieties. Total soluble sugar was decrease as compared to their control chili group with − 252.49%, -239.40% -232.87%, -218.37%, -207.13%, -158.90%, -151.51% in V6, V2, V4, V3, V1, V7 and V5 at 5 mg Cd/kg soil application.

Flavonoid content was significantly decrease in all *Capsicum annuum* L. varieties against Cd stress at all levels. The least trend of flavonoid content was found in Desi and G-916 varieties. The maximum flavonoids among varieties were in V5 (BR-763), followed by V6 (BG-912), V7 (F1-9226), V3 (Sathra) and V1 (Hybrid). Flavonoids content was decrease with − 37.63% (Sathra), -34.78% (Hybrid), -33.85% (G-916), -31.96% (F1-9226), -31.44% (Desi), -30.58% (BR-763), -22.88% (BG-912) as compared to their control at 5 mg Cd/kg stress.

### MDA and H_2_O_2_ content

**Fig. 5:**
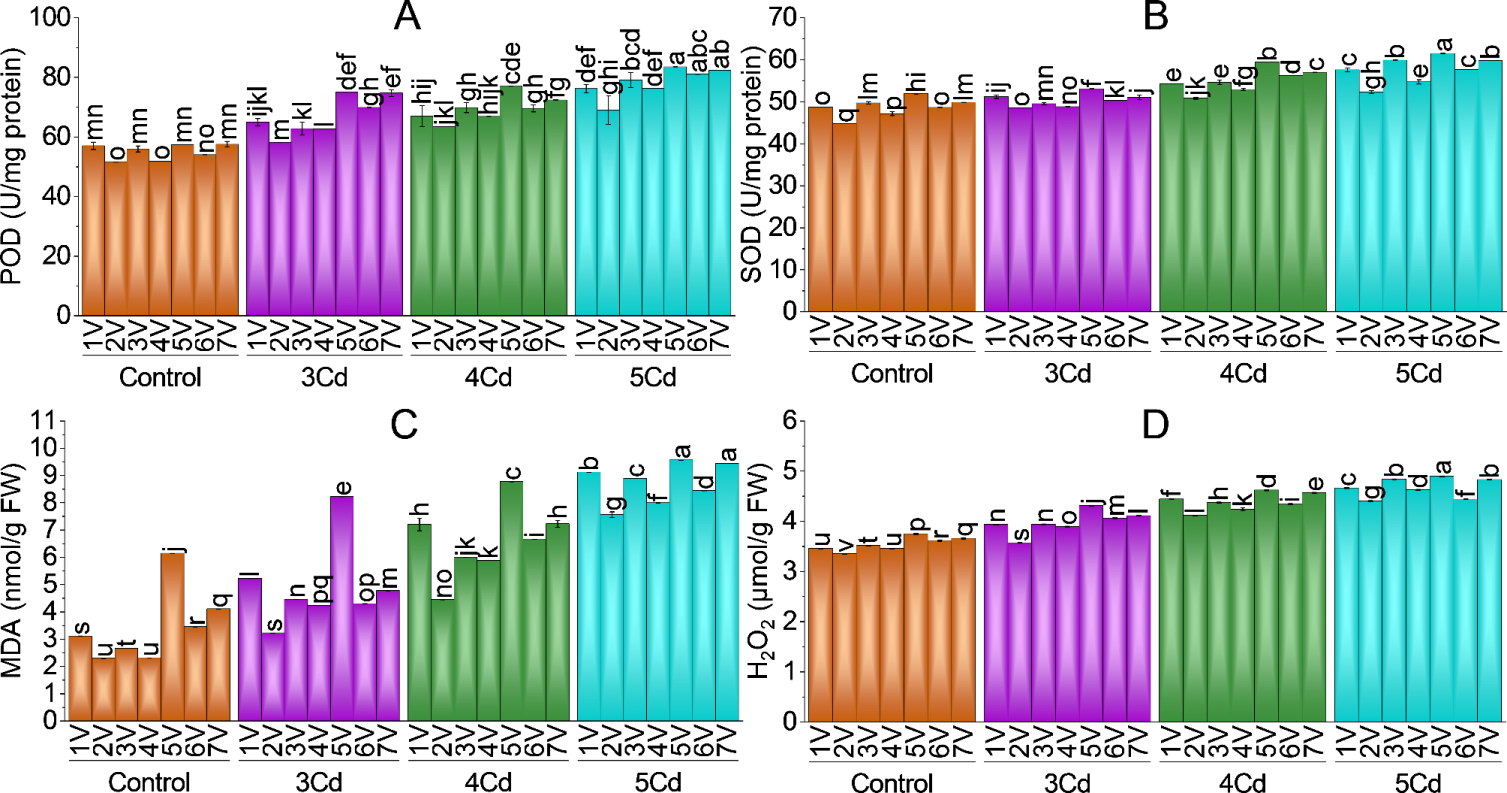
Effect of variable toxicity levels of Cd on POD (**A**), SOD (**B**), MDA (**C**) and H_2_O_2_ (**D**) of different chili varieties. Bars are means ± SE of 3 replicates. Different bar letters showed significant changes at *p* ≤ 0.05; Fisher’s LSD

Lipid oxidation markers (malondialdehyde and hydrogen peroxide content) were significantly increased in all Capsicum varieties against Cd stress as compared to the control group (Fig. 5-C & D). Maximum and almost similar MDA and H_2_O_2_ content was found in V5 (BR-763), V6 (BG-912), V1 (Hybrid) and V3 (Sathra) among all chili varieties. MDA and H_2_O_2_ were the least in V2 (Desi) and V4 (G-916) capsicum varieties. In comparison with their control group, the maximum decrease in MDA at 5 mg Cd/kg stress was − 50.01% (V6), followed by -47.16% (V4), -45.48% (V5), -43.11% (V7) -41.38% (V3), -33.81% (V2) and − 33.66% (V1). Hydrogen peroxide content in response to oxidative stress also decrease with the trend of -20.53%, -19.89%, -18.58%, -18.50%, -18.23%, 16.85%, and 16.10% in chili variety Sathra, F1-9226, BR-763, BG-912, Hybrid, Desi and G-916 as compared to control plants.

### Antioxidants

Cadmium-stressed chili plants had higher antioxidants (peroxidase, superoxide dismutase, catalase, and ascorbate peroxidase) than control plants. The maximum decrease in POD, SOD and CAT was − 31.81%, -25.98%, -16.39% in chili variety V7 (F1-9226) at 5 mg Cd/kg stress compared to its control. While maximum APX content decrease was − 82.91% followed by -80.16%, -65.19%, -40.31%, -30.14%, -10.34% and − 6.45% in V4 (G-916), V2 (Desi), V3 (Sathra), V6 (BG-912), V1 (Hybrid), V7 (F1-9226) and V5 (BR-763) at 5 mg Cd/kg treatment as compared to control chili plants. POD and SOD were significantly increased in all *Capsicum* varieties (Fig. 5A & B). Least POD and SOD activity was found in V2 (Desi) and V4 (G-916) as they tolerated Cd stress more. V5, V3, V1 and V7 (BR-763, Sathra, Hybrid and F1-9226) have maximum POD and SOD content as they were more susceptible to Cd stress.

**Fig. 6:**
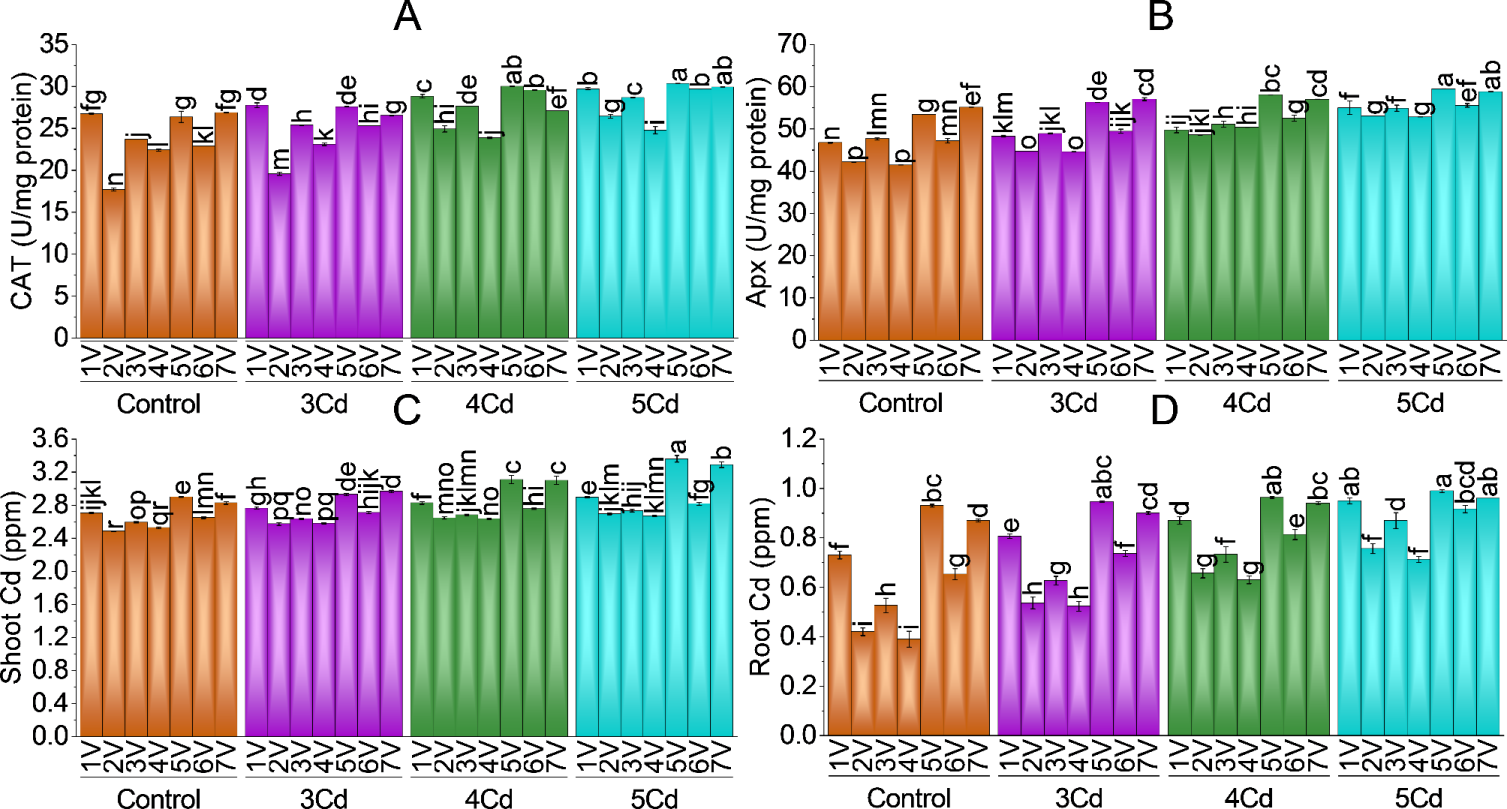
Effect of variable toxicity levels of Cd on CAT (**A**), Apx (**B**), shoot Cd (**C**) and root Cd (**D**) of different Capsicum varieties. Bars are means ± SE of 3 replicates. Different bar letters showed significant changes at *p* ≤ 0.05; Fisher’s LSD

Catalase and ascorbate peroxidase activity have the same trend as peroxidase and superoxide dismutase (Fig. 6A & B). Compared to untreated plants, a significant increase in catalase and ascorbate peroxidase was found in all chili varieties with increasing Cd. Maximum CAT was found in 5 chili varieties except Desi and G-916.

### Cadmium accumulation in shoot and root

A rising trend of Cd accumulation was observed both in root and shoot in chili varieties with the increased levels of Cd in the soil (Fig. 6C & D). root comparatively accumulated lesser quantities of Cd than shoot in all seven *Capsicum* varieties. The Cd percent increase accumulation was higher in V5 (BR-763) than in the other seven varieties at all Cd levels. In shoot and root, the least Cd was accumulated in V2, V3, V4 and V6 (Desi, Sathra, G-916 and Bg-912) at all levels of Cd treatment. V1, V5 and V7 (Hybrid, BR-763 and F1-9226) have maximum Cd accumulation in both root and shoot.

### Translocation factor and tolerance index in capsicum varieties

**Fig. 7:**
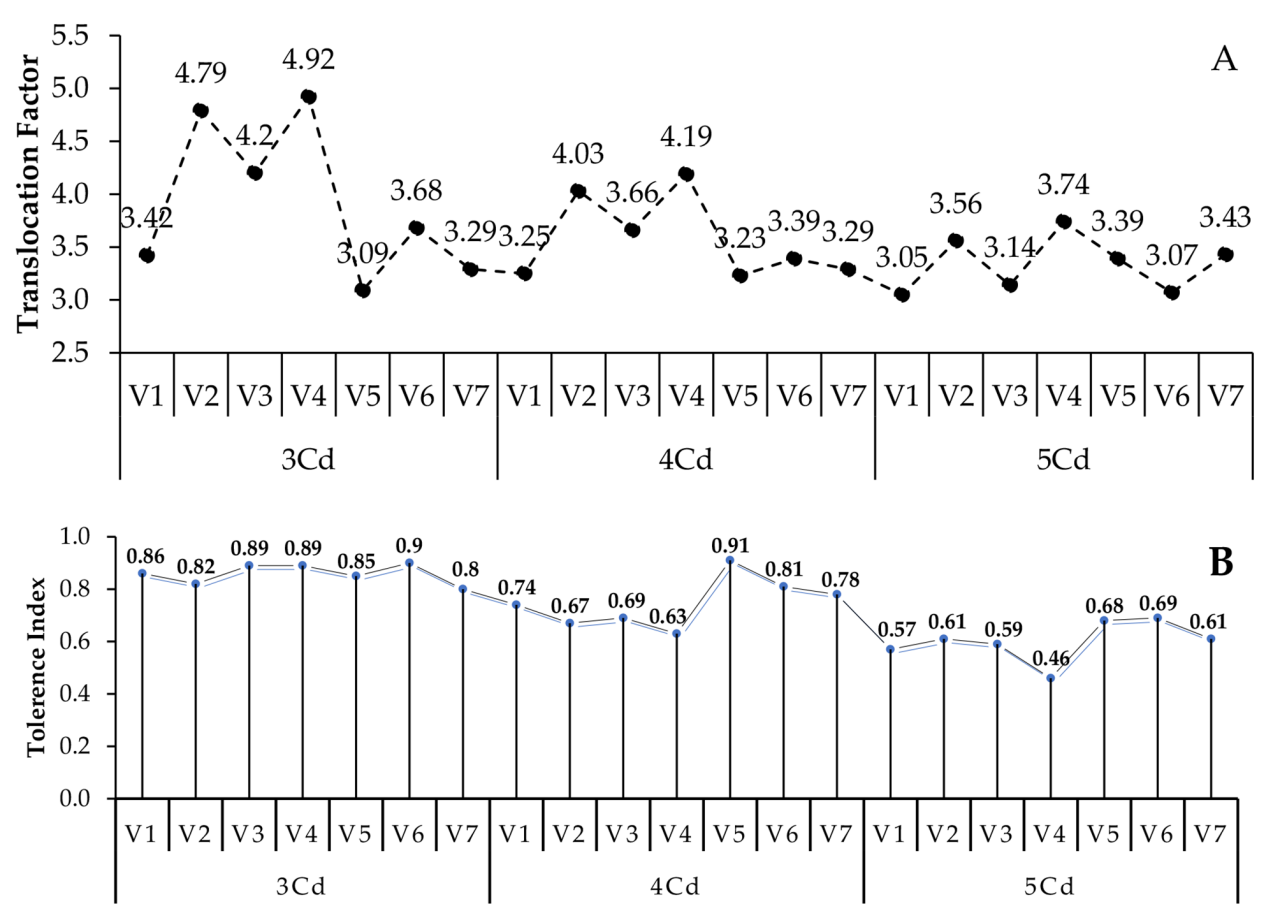
Translocation Factor (**A**) and Tolerance Index (**B**) of different chili varieties. Lines are means ± SE of 3 replicates

Data from Table 2 indicates that the Translocation Factor (TF) was described as the ratio of Cd in the shoot to that in the root of chili varieties. The translocation factor was found in the order of V4 (G-916) > V2 (Desi) > V3 (Sathra) > V6 (BG-912) > V1 (Hybrid) > V7 (F1-9226) and > V5 (BR-763) at 3, 4 and 5 mg Cd/kg application in all chili varieties. Maximum translocation factor was 4.93, 4.18 and 3.74 in variety 4 (G-916) at 3, 4 and 5 mg Cd/kg stress (Fig. 7-A).

The Tolerance Index (TI) of chili varieties in root length attribute was highest in V6 (BG-912), followed by V5 (BR-763), V2 (Desi), V7 (F1-9226), V3 (Sathra), V1 (Hybrid) and V4 (G-916) (Table 3). All these varieties showed preferable tolerance to Cd stress (Fig. 7-B).

**Table 2:** Translocation Factor in root length for Cd stress in chili varieties

S. No	Chili Varieties	Translocation Factor			Feasibility of the Chili varieties for the Phytoremediation of Cadmium
		3 mg Cd/kg soil	4 mg Cd/kg soil	5 mg Cd/kg soil	
1	V1 (Hybrid)	3.43	3.25	3.05	Cannot be used for the metal remediation.
2	V2 (Desi)	4.79	4.03	3.56	
3	V3 (Sathra)	4.20	3.66	3.14	
4	V4 (G-916)	4.93	4.18	3.74	
5	V5 (BR-763)	3.09	3.22	3.39	
6	V6 (BG-912)	3.68	3.39	3.07	
7	V7 (F1-9226)	3.29	3.29	3.42	

**Table 3:** Tolerance Index in root length for Cadmium stress in chili varieties

S. No	*Capsicum annuum* L. Varieties	Tolerance Index of Root Length		
		3 mg Cd/kg soil	4 mg Cd/kg soil	5 mg Cd/kg soil
1	V1 (Hybrid)	0.86	0.74	0.57
2	V2 (Desi)	0.82	0.67	0.61
3	V3 (Sathra)	0.89	0.69	0.59
4	V4 (G-916)	0.75	0.63	0.46
5	V5 (BR-763)	0.85	0.81	0.68
6	V6 (BG-912)	0.91	0.82	0.69
7	V7 (F1-9226)	0.80	0.78	0.61

### Principal component analysis

**Fig. 8:**
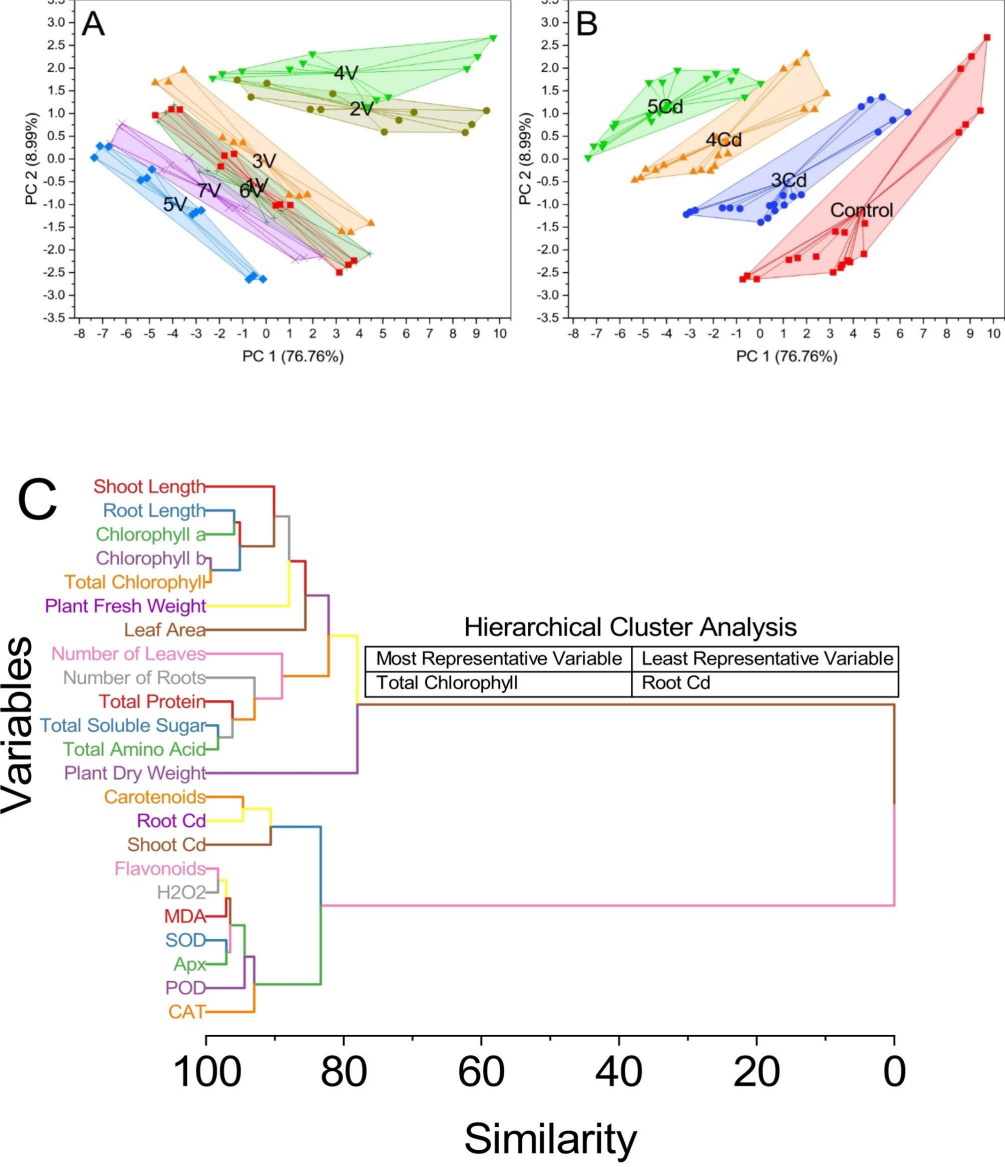
Principal component analysis of *Capsicum annuum* L. variety vise (**A**), Cadmium treatment vise (**B**) and hierarchical cluster analysis (**C**) of morphological, physiological, and biochemical attributes

PC1 explains 76.76% of the total variation, while PC2 explains 8.99%. The samples were labeled based on their scores in the PC1 and PC2 spaces. The “Control” group, represented by scores 3.77057 and − 2.23502, was clustered with samples displaying scores 3.5242 and − 2.32787 and scores 3.1354 and − 2.49455. Another group labeled “3Cd” consisted of samples with scores 1.04039 and − 1.0139, scores 0.60498 and − 1.0003, and scores 0.41661 and − 1.02538. Similarly, the “4Cd” group included samples with scores − 1.36774 and 0.11357, scores − 1.78588 and 0.073, and scores − 1.93724 and − 0.1628. Lastly, the “5Cd” group comprised samples with scores − 3.69152 and 1.08801, scores − 4.05324 and 1.09506, and scores − 4.74804 and 0.96117 (Fig. 8-A). Additionally, the “Control” group displayed samples with scores 9.44672 and 1.06415, scores 8.81442 and 0.76037, and scores 8.52901 and 0.58291. The “3Cd” group included samples with scores 6.33798 and 1.02849, scores 5.6899 and 0.85463, and scores 5.0641 and 0.59455. The “4Cd” group consisted of samples with scores 2.85689 and 1.43604, scores 2.34291 and 1.08783, and scores 1.89495 and 1.09338. Lastly, the “5Cd” group contained samples with scores 0.03588 and 1.66646, scores − 0.64971 and 1.36237, and scores − 1.23677 and 1.73804. The cluster plot further revealed the grouping of samples within the defined convex hulls. The “Control” group exhibited samples with scores 4.49269 and − 1.41648, scores 3.62868 and − 1.61612, and scores 3.23445 and − 1.59685, while the “3Cd” group included samples with scores 1.7719 and − 0.78618, scores 1.41788 and − 0.82296, and scores 0.9914 and − 0.80009. The “4Cd” group comprised samples with scores − 0.99093 and 0.35708, scores − 1.39687 and 0.3613, and scores − 1.86509 and 0.46662. Lastly, the “5Cd” group displayed samples with scores − 3.53312 and 1.95242, scores − 4.17407 and 1.69037, and scores − 4.75127 and 1.67755.

In the plot, the samples labeled as 1 V (represented by scores 3.77057 and − 2.23502, scores 3.5242 and − 2.32787, and scores 3.1354 and − 2.49455) were clustered together, indicating their similarity in the PC space (Fig. 8-B). Similarly, samples labeled as 2 V (with scores 9.44672 and 1.06415, 8.81442 and 0.76037, and 8.52901 and 0.58291) formed a distinct cluster. The samples labeled as 3 V (with scores 4.49269 and − 1.41648, scores 3.62868 and − 1.61612, and scores 3.23445 and − 1.59685) were also clustered together, indicating their similarity. The samples labeled as 4 V (with scores 9.73178 and 2.67227, scores 9.06212 and 2.25583, and scores 8.60333 and 1.98806) formed another distinct cluster. Samples labeled as 5 V (with scores − 0.13444 and − 2.64219, scores − 0.53998 and − 2.56739, and scores − 0.7357 and − 2.64918) were clustered together, as well as samples labeled as 6 V (with scores 4.45716 and − 2.09014, scores 3.86352 and − 2.26906, and scores 3.46249 and − 2.39371). Similarly, samples labeled as 7 V (with scores 2.40914 and − 2.14693, scores 1.61846 and − 2.17758, and scores 1.24508 and − 2.22007) formed a separate cluster. The hierarchical cluster plot analysis was conducted to investigate the similarities between variables in the dataset (Fig. 8-C). The results revealed distinct clusters based on the proximity of variables. Cluster 1 consisted of variables 4 and 29, representing chlorophyll b and Total Chlorophyll, with a similarity of 0.65695. Additionally, variables 17 and 24 (Flavonoids and H_2_O_2_) exhibited a similarity of 1.74058. In contrast, variables 11 and 27 (Total Soluble Sugar and Total Amino Acid) demonstrated a similarity of 1.74167. Cluster 2 comprised variables 19 and 25 (MDA), with a similarity of 2.92504, along with variables 20 and 26 (SOD and APX), displaying a similarity of 2.93947. Moving on to Cluster 3, variables 10 and 32 (Total Protein) demonstrated a higher similarity of 3.82237. In contrast, variable 27 had a similarity of 2.0807 with the rest of the variables in this cluster. Cluster 4 showed variables 2 and 28 (Root Length and Chlorophyll a) with a similarity of 4.08382. Furthermore, variables 28 and 34 exhibited a similarity of 0.81849, and variables 29 and 34 had a similarity of 4.24536. In Cluster 5, variables 14 and 33 (Carotenoids and Root Cd) demonstrated a similarity of 5.35159, while variables 30 and 31 had a similarity of 2.0939. Additionally, variables 22 and 31 (POD) showed a higher similarity of 5.59838. Cluster 6 involved variables 31 and 39 with a similarity of 1.39293, and variables 23 and 39 (CAT) exhibited a higher similarity of 6.99131. Cluster 7 comprised variables 9 and 35 (Number of Roots) with a similarity of 7.04184, whereas variable 32 showed a similarity of 3.21947 with the rest of the variables in this cluster. In Cluster 8, variables 33 and 38 demonstrated a similarity of 4.05485, and variables 16 and 38 (Shoot Cd) exhibited a higher similarity of 9.40645. Cluster 9 included variables 1 and 36 (Shoot Length) with a similarity of 9.88385, while variables 34 and 36 showed a similarity of 4.98154. Cluster 10 consisted of variables 8 and 41 (Number of Leaves) with a similarity of 11.03897, and variable 35 displayed a similarity of 3.99713 with the rest of the variables in this cluster. Moving on to Cluster 11, variables 6 and 37 (Plant Fresh Weight) exhibited a similarity of 12.06472, while variables 37 and 40 had a similarity of 2.35611. Cluster 12 involved variables 7 and 40 (Leaf Area) with a higher similarity of 14.42083. In Cluster 13, variables 38 and 44 showed a similarity of 7.24966, and variables 39 and 44 demonstrated a higher similarity of 9.6648. Cluster 14 included variables 40 and 42 with a similarity of 3.38224, and variables 41 and 42 exhibited a similarity of 6.76409. Cluster 15 involved variables 42 and 43 with a similarity of 4.17401, while variable 13 (Plant Dry Weight) displayed a remarkably higher similarity of 21.97708 with the rest of the variables in this cluster. Finally, variables 43 and 45 demonstrated similarities of 78.02292 and 83.34389, respectively, with variable 45 representing a separate cluster.

**Fig. 9:**
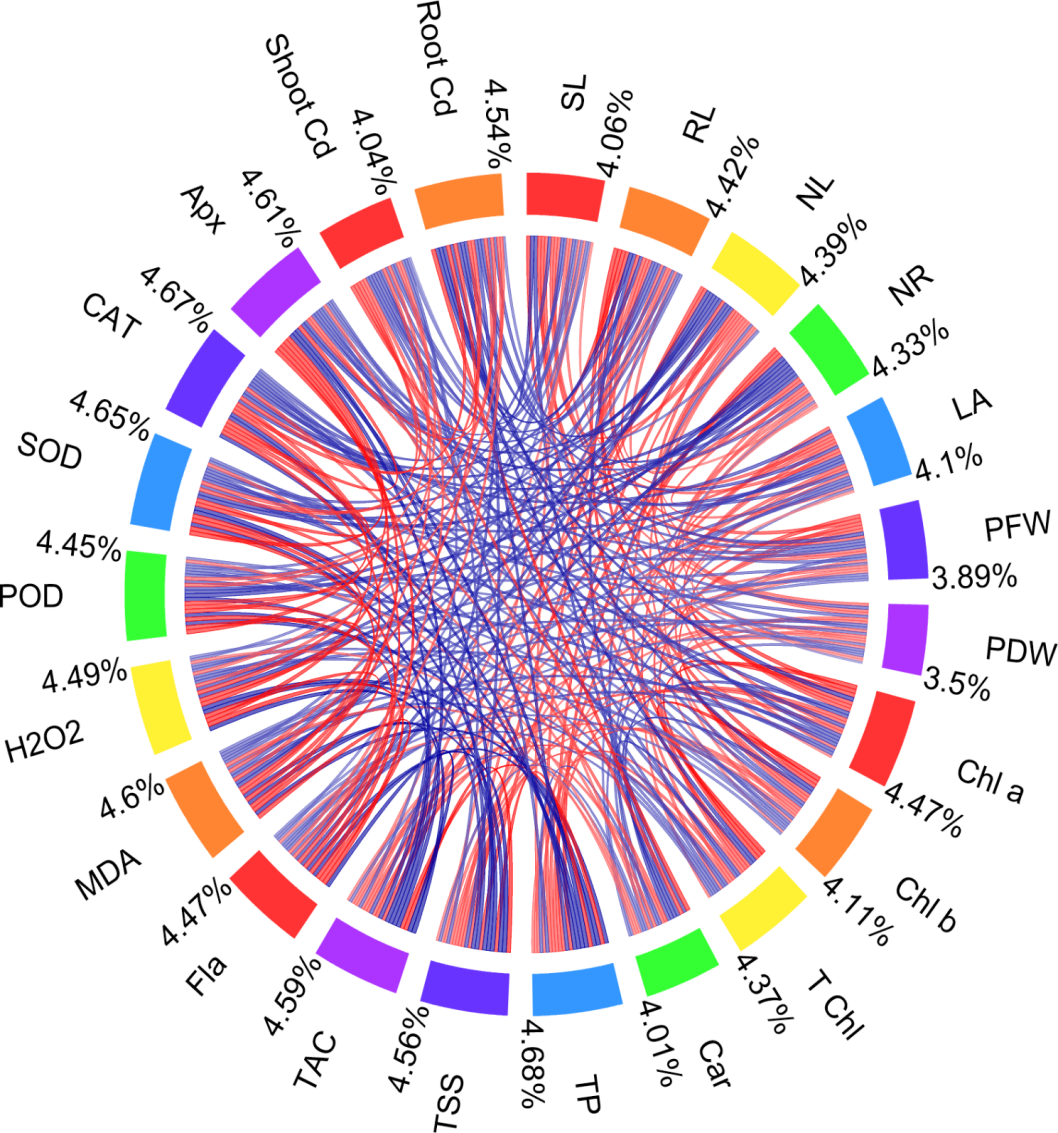
Correlation chord plot for morphological, physiological, and biochemical attributes in 7 chili varieties

The correlation coefficients from the correlation chord plot are presented in the following paragraph (Fig. 9). The absolute values of the correlation coefficients (|C|) are provided to indicate the strength of the correlation. There were several positive correlations observed. Notable positive correlations include SL with RL (|C| = 0.9046), NL (|C| = 0.73335), Chl a (|C| = 0.82363), and T Chl (|C| = 0.81104). Additionally, RL exhibited strong positive correlations with NL (|C| = 0.76193), Chl a (|C| = 0.9289), and T Chl (|C| = 0.93072). On the other hand, there were several negative correlations observed as well. Notable negative correlations include Car with SL (|C| = 0.83754), RL (|C| = 0.81996), and NL (|C| = 0.66984). Moreover, Shoot Cd exhibited strong negative correlations with Car (|C| = 0.85056), SL (|C| = 0.69626), RL (|C| = 0.72079), and NL (|C| = 0.8046). Other noteworthy correlations include PFW with SL (|C| = 0.74508) and RL (|C| = 0.8194), Chl b with SL (|C| = 0.77238) and RL (|C| = 0.90039), and T Chl with SL (|C| = 0.81104) and RL (|C| = 0.93072).

## Discussion

One of the most hazardous heavy metals, Cd, threatens food security and crop productivity [46]. Chlorosis, slowed development, reduced photosynthesis, and ultrastructural damage are all effects of Cd poisoning on plants [47]. Additionally, Cd may interfere with the absorption of other mineral nutrients such as Fe, Ca, Zn, and Mn, leading to an unbalanced diet [48]. Plant genotypes differ in their capacity to take up and move Cd that has been altered in the soil from the roots to the shoots [49]. One of the most notable aspects of Cd toxicity in plants is a reduction in growth metrics such as tomato [50] cosmos [51], wheat [52] and cucumber [53]. The present study was conducted to evaluate the tolerant and susceptible chili varieties against Cd stress. Cadmium causes an overall reduction in the seven chili varieties’ growth attributes. Our results showed that V2 (Desi) and V4 (G-916) are more tolerant to Cd stress. At the same time, V3 (Sathra) and V7 (F1-9226) are intermediate and V1 (Hybrid), V5 (BR-763), and V6 (BG-912) are susceptible.

In this study, the application of 3, 4, and 5 mg Cd/kg treatment to seven chili varieties causes damage to morphological, physiological, and biochemical properties compared to untreated plants. Shoot length, root length, plant fresh weight, plant dry weight, leaf area, number of leaves, and roots were decreased at all Cd levels to chili plants. Reducing chili plant’s fresh and dry weight against Cd stress follows [51], where Cd has a deleterious impact *Zea mays* fresh and dry weight. These results are similar to [54] where Cd has impaired plant dry weight accumulation. Decreased chili weight at maximum Cd stress level (5 mg Cd/kg in soil) is similar in bitter gourd, with a significant decrease in fruit length, fresh weight, and yield per plant [55]. Another study has similar results to ours, where Cd treatment in *Brassica napus* inhibited growth and biomass [56]. The decrease in leaf area, fresh and dry mass, and root and shoot length was also mediated by Cd [57]. After applying Cd, Jiang et al. [56] discovered a significant decrease in garlic plant’s plant biomass and leaf area. Panković et al. [58] documented that Cd inhibitory effects improved sunflower’s nitrogen uptake. With rising Cd concentrations, a persistent trend of decreased shoot and root length was found in chili crop plants [59]. Cadmium toxicity inhibits plant growth, reducing root dry weight, diameter, and the number of lateral roots in Massai grass [60].

Cadmium stress reduces the efficiency of chlorophyll contents (chlorophyll a, chlorophyll b, and total chlorophyll) in *Capsicum annuum* L. Cd is also responsible for a decrease in photosynthetic pigments and PSII efficiency in *C. annuum* L. cultivars, as well as a decrease in energy transfer from chlorophyll an antenna connected to the PSII light-harvesting complex to the reaction center [61]. Sun et al. [62] observed that Cd has a deleterious impact on the growth and photosynthetic pigments of *Solanum nigrum* L., except for antioxidants. In a study, Chen et al. [63] found that at 24 mg Cd/kg soil, all of the photosynthetic pigments of mustard (*Brassica juncea*) and pakchoi (*Brassica campestris*) were reduced. Results by Ali et al. [64] in *Brassica juncea* plants complement our findings that photosynthetic pigments were reduced under Cd and Pb stress. These reductions are primarily the result of Cd toxic action on chlorophyll, which leads to the destruction of Rubisco and the disintegration of chlorophyll molecules [65, 66]. Cadmium has a deleterious impact on the contents of chlorophyll a, b, and total chlorophyll in tomato plants [67]. By lowering plant’s ability to absorb magnesium, Cd prevents chlorophyll production in leaves, reducing photosynthesis in maize cultivars [68]. An increase in accessory pigment carotenoids was found in our results. The highest concentrations of carotenoids were found in the V1 (Hybrid), V5 (BR-763), V7 (F1-9226), and other variants under investigation. According to Gubrelay et al. [69] the benefits of Cd on carotenoids grow with concentration. Under severe Cd stress and a combination of citric acid, an increase in chlorophyll and carotenoids was seen in *Brassica napus* [70].

Total proteins, total amino acids, and total soluble sugars were decreased in all Cd-treated chili varieties compared to their controls. Our findings are similar to [71] where proteins were decreased against Cd stress in tomato crops. At the same time, flavonoids were increased in all varieties with maximum V5 (BR-763) at 5 mg Cd/kg application. These results are like the study of *Robinia pseudoacacia* L. seedlings against Cd stress [72]. To reduce the oxidative activities of ROS in plants growing in Cd-contaminated soils, flavonoids possess hydroxyl or carboxyl groups that can chelate Cd [73–75]. Cadmium causes an excess of ROS to be produced, oxidative stress to be caused in essential cellular components, including lipids and proteins, and an imbalance in plant metabolism [76].

Once Cd exceeds the threshold, enzyme activity is affected or even decreases, resulting in the inability of plants to remove malonaldehyde and other peroxides and causing plant poisoning. In *Dendrobium officinale* seedlings and *Thymus vulgaris* seeds, Cd significantly induced malondialdehyde and proline accumulation [77]. As a direct secondary effect of H_2_O_2_ and O_2_ buildup brought on by Cd stress, membranes, proteins, and nucleic acids might suffer damage [78]. Malondialdehyde and hydrogen peroxide content were significantly increased in all *Capsicum* varieties against Cd stress as compared to the control group (Fig. 5-C & D). Maximum and almost similar MDA and H_2_O_2_ content was found in V5 (BR-763), V6 (BG-912), V1 (Hybrid) and V3 (Sathra) among all chili varieties. The root’s relative length and malondialdehyde level increase indicate Cd toxicity [79]. The potato leaves’ MDA level rose in response to Cd exposure due to increased membrane lipid peroxidation [80].

Activating the antioxidant defense system, reducing Cd uptake and accumulation, sequestering Cd into vacuoles, chelating Cd in the cytosol through various ligands, and precipitating Cd in the cell wall are just a few of the several defense mechanisms that plants have evolved to combat Cd toxicity [81]. Plants increased their production of antioxidants to resist the stress caused by heavy metals [82]. In this study, peroxidases, superoxide dismutases, catalases, and ascorbate peroxidases were all increased in *Capsicum annuum* L. varieties as a defensive response against Cd stress compared to control. Maximum antioxidants were increased at 5 mg Cd/kg soil application compared to other treatment levels. The higher activity of antioxidant enzymes offers larger detoxifying effectiveness, which improves a plant variety’s resistance to heavy metal-induced oxidative stress, as demonstrated by [83] in *Brassica juncea.* Cadmium application increased antioxidant activities, a stress-defensive response in tobacco plants [84]. *Vigna mungo* seedlings showed an increased number and intensity of peroxidases during plant growth and development when Cd^2+^ was administered singly or in combination [85] similar to our results. The findings of Shaw [86] indicated that increased Cd levels enhanced POD activity. According to research by Ferreira et al. [87], heat stress and exposure to Cd in higher plants are known to promote the expression of cytosolic Cu/Zn-SOD. Increased expression of the genes encoding these enzymes may cause the increased SOD activity in the chili plant. Similar outcomes were also noted in some crops under heavy metal stresses, such as Cd stress in *Hibiscus cannabinus* [88]. According to Romero-Puertas et al. [89], Cd can influence the expression and regulation of the superoxide dismutase isoenzyme. According to Gill & Tuteja [90], catalases are tetrameric, heme-containing enzymes that catalyze the dismutation of H_2_O_2_ into oxygen and water. *Oryza sativa* [91], barley root [92] and heavy metal treatments showed increased CAT activity, allowing active scavenging of H_2_O_2_. Similar catalase augmentation results for *Oryza sativa*, *Brassica juncea*, and *Solanum lycopersicum* plants were found by [93–95]. Guo et al. [96] studied cucumbers under Cd stress and found that stress activates genes related to ROS scavenging, which in turn increases the activity of the enzymes APX, SOD, CAT, and GR as well as GSH content, leading to a significant reduction in H_2_O_2_ and O_2_ levels. According to Nahakpam & Shah [97], increased APX activity in plants under Cd^2+^ stress indicates that the ascorbate-glutathione cycle has been activated in rice plants. In response to Cd exposure, APX enzymes are elevated in rice cultivars [98].

In all seven varieties of Capsicum, the shoot accumulated more Cd than the root on a relative basis. V5 (BR-763) had a larger percentage increase in Cd accumulation at all Cd levels than the other seven types. These results are similar to Huang et al. [99], who reported that Cd is more sequestered in the shoot than in the root in *Oryza sativa* plants. In another study, less Cd was translocated to the upper parts in micro-tom plants when compared to Never-ripe *and* Diageotropica tomato *shoots* [100].

The translocation factor was the maximum in V4 (G-916) and least in V5 (BR-763) at all levels of Cd treatment among the studied seven chili varieties. The tolerance index for root length was highest in V6 (BG-912) compared to other chili varieties against Cd stress. Tolerance is another trait that affects the process of phytoremediation [101]. Suppose the tolerance index is less than 1. In that case, this means that metal contamination has put the plant under stress, resulting in a net loss of biomass. In contrast, tolerance index values larger than 1 denote the development of plant tolerance and a net increase in biomass (hyperaccumulator) [102].

## Conclusion

This study evaluated the tolerant, susceptible, and intermediate *Capsicum annuum* L. varieties against Cd stress (3, 4, and 5 mg Cd/kg) were spiked in loamy soil, causing the reduction in chlorophyll contents (chlorophyll a and b), shoot length, root length, plant biomass, total proteins, amino acids, and soluble sugars in all seven *Capsicum* varieties. Cadmium exposure causes a significant increase in flavonoids, MDA, hydrogen peroxide, and antioxidant (POD, CAT, SOD and APX) levels in all varieties. Enhanced activity of antioxidants and flavonoid content has protected chili varieties. V2 (Desi) and V4 (G-916) showed maximum growth among their controls, compared with all other varieties termed most tolerant against Cd stress.

### Electronic supplementary material

Below is the link to the electronic supplementary material.

**Supplementary Material 1: Figure S1:** Cultivated chili varieties (i.e., V1 (Hybrid) = A, V2 (Desi) = B, V3 (Sathra) = C, V4 (G-916) = D, V5 (BR-763) = E, V6 (BG-912) = F and V7 (F1-9226) = G) grown under cadmium stress. **Figure S2:** Comparison of control (A) and Cd stress (B) plants

## Data Availability

All data generated or analyzed during this study are included in this published article.

## Declarations

### Study protocol must comply with relevant institutional, national, and international guidelines and legislation

Our experiment follows the relevant institutional, national, and international guidelines and legislation.
