# Supplementary material for: Differential responses of chili varieties grown under cadmium stress
Source: BMC Plant Biol. 2024 Jan 2;24:7. doi: 10.1186/s12870-023-04678-x (PMC10759427; doi:10.1186/s12870-023-04678-x)
Supplement: Supplementary file 1 — Supplementary Material 1: Figure S1: Cultivated chili varieties (i.e., V1 (Hybrid) = A, V2 (Desi) = B, V3 (Sathra) = C, V4 (G-916) = D, V5 (BR-763) = E, V6 (BG-912) = F and V7 (F1-9226) = G) grown under cadmium stress. Figure S2: Comparison of control (A) and Cd stress (B) plants [file 12870_2023_4678_MOESM1_ESM.docx]

A
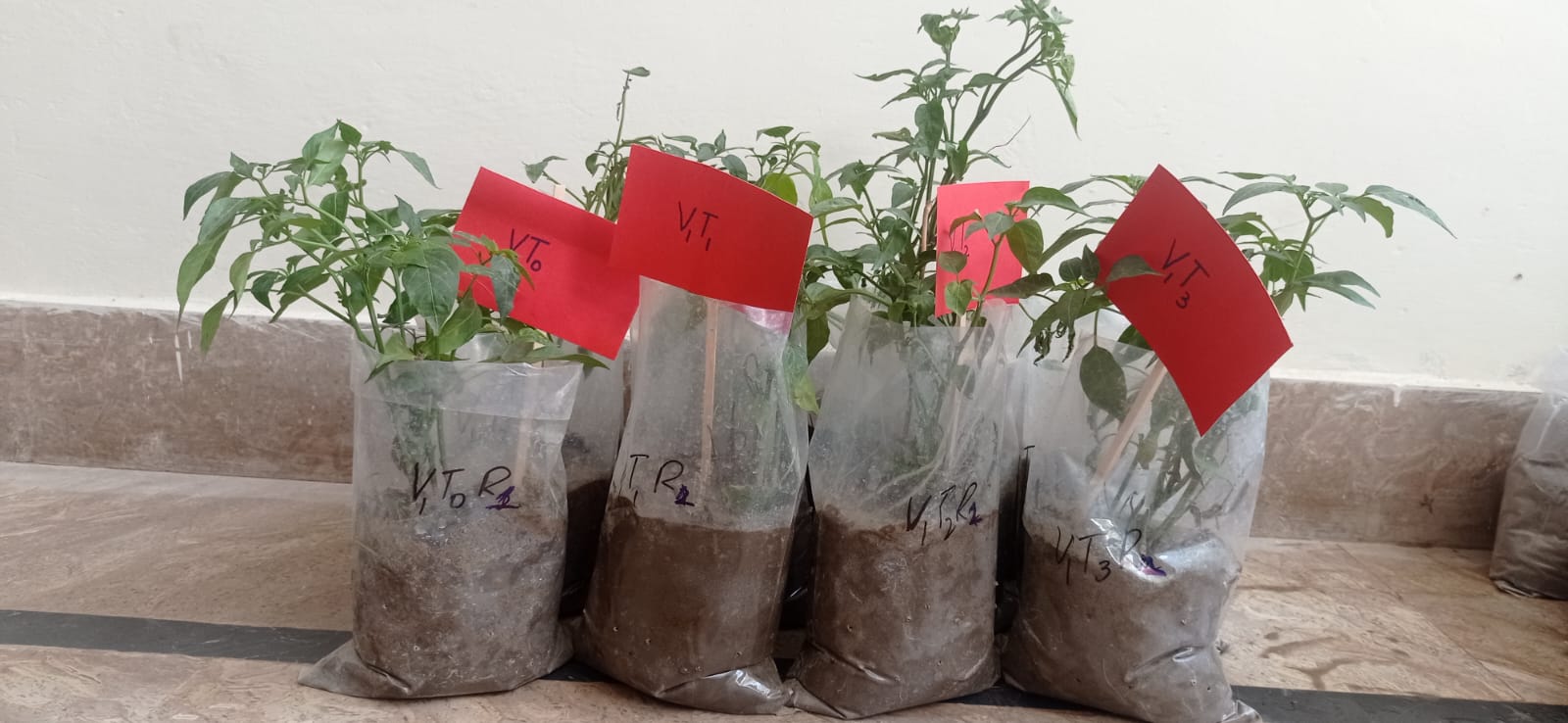


B
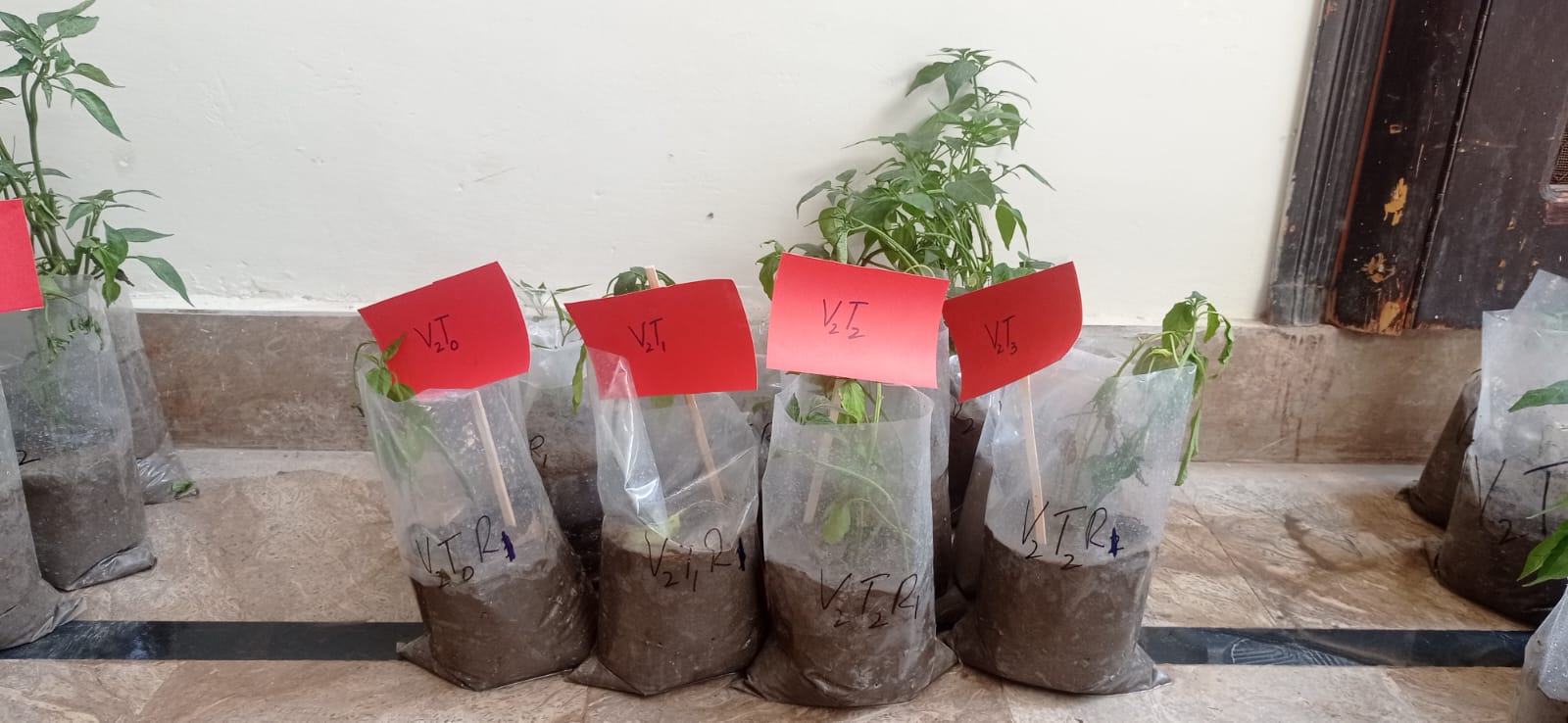


C
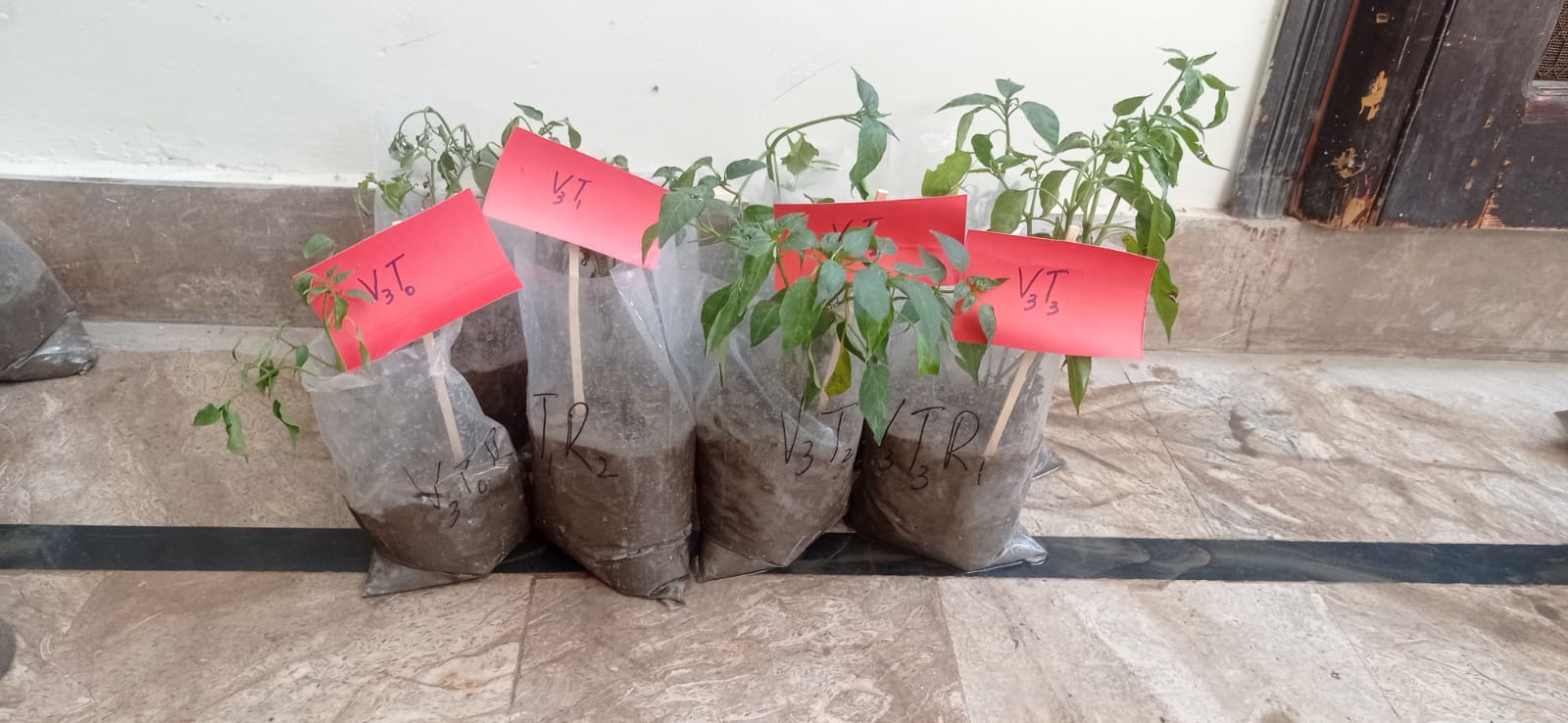


D
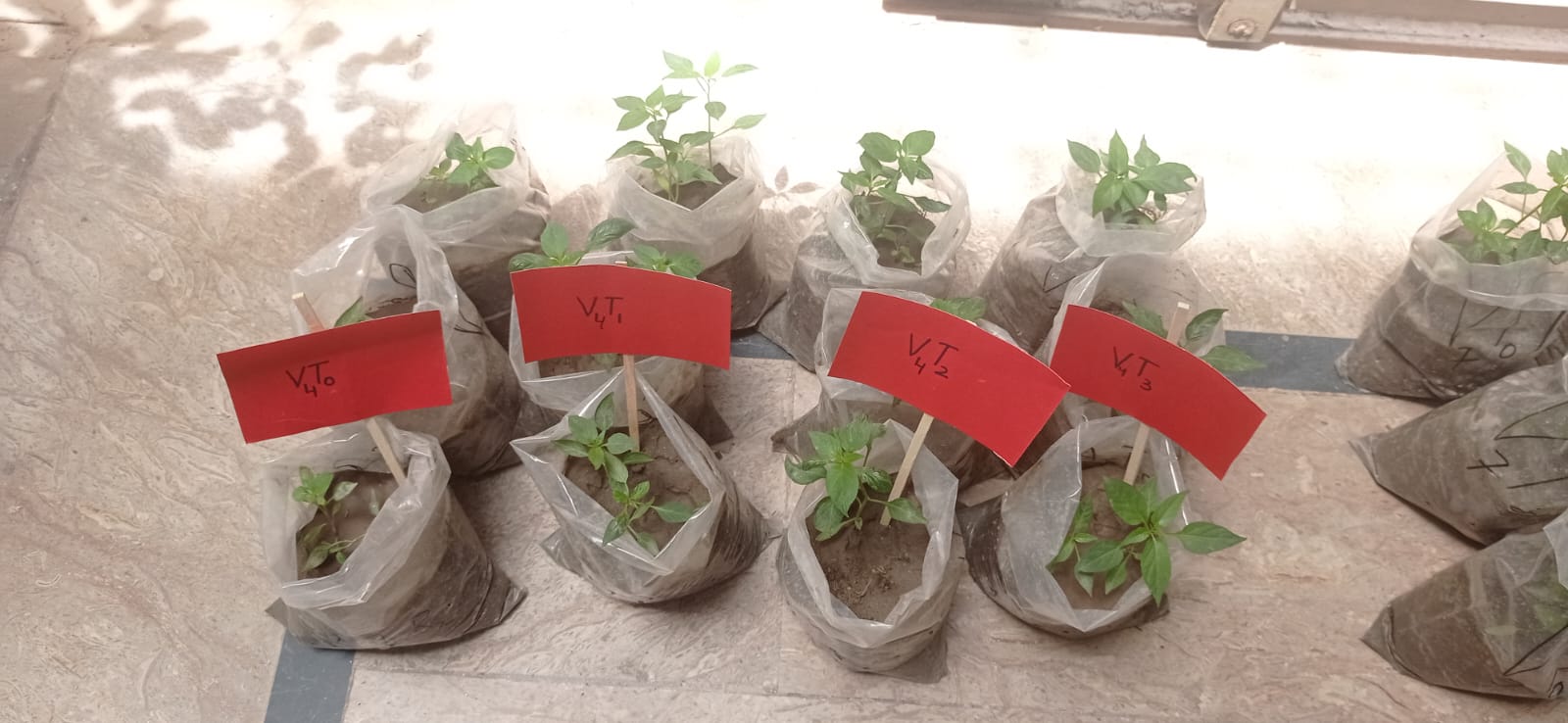


E
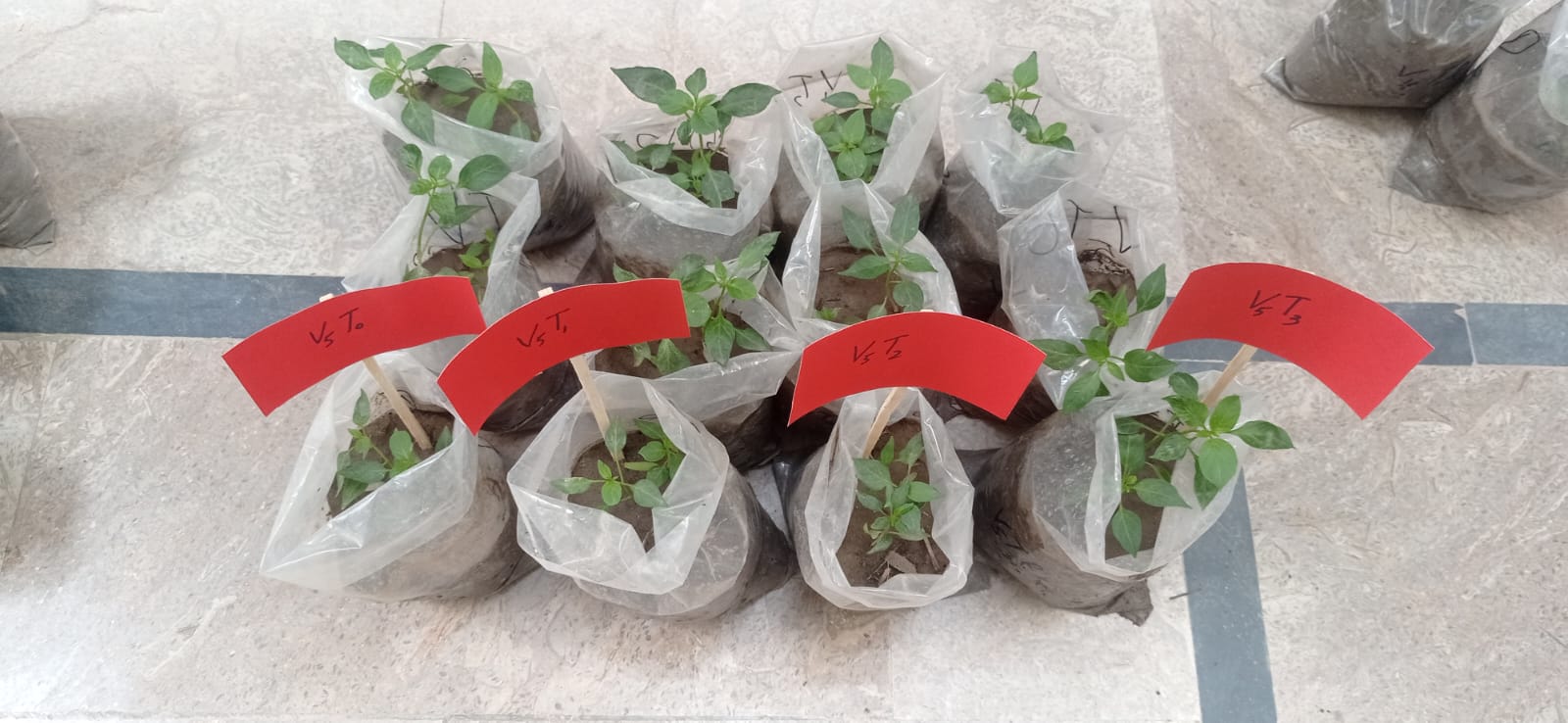

F
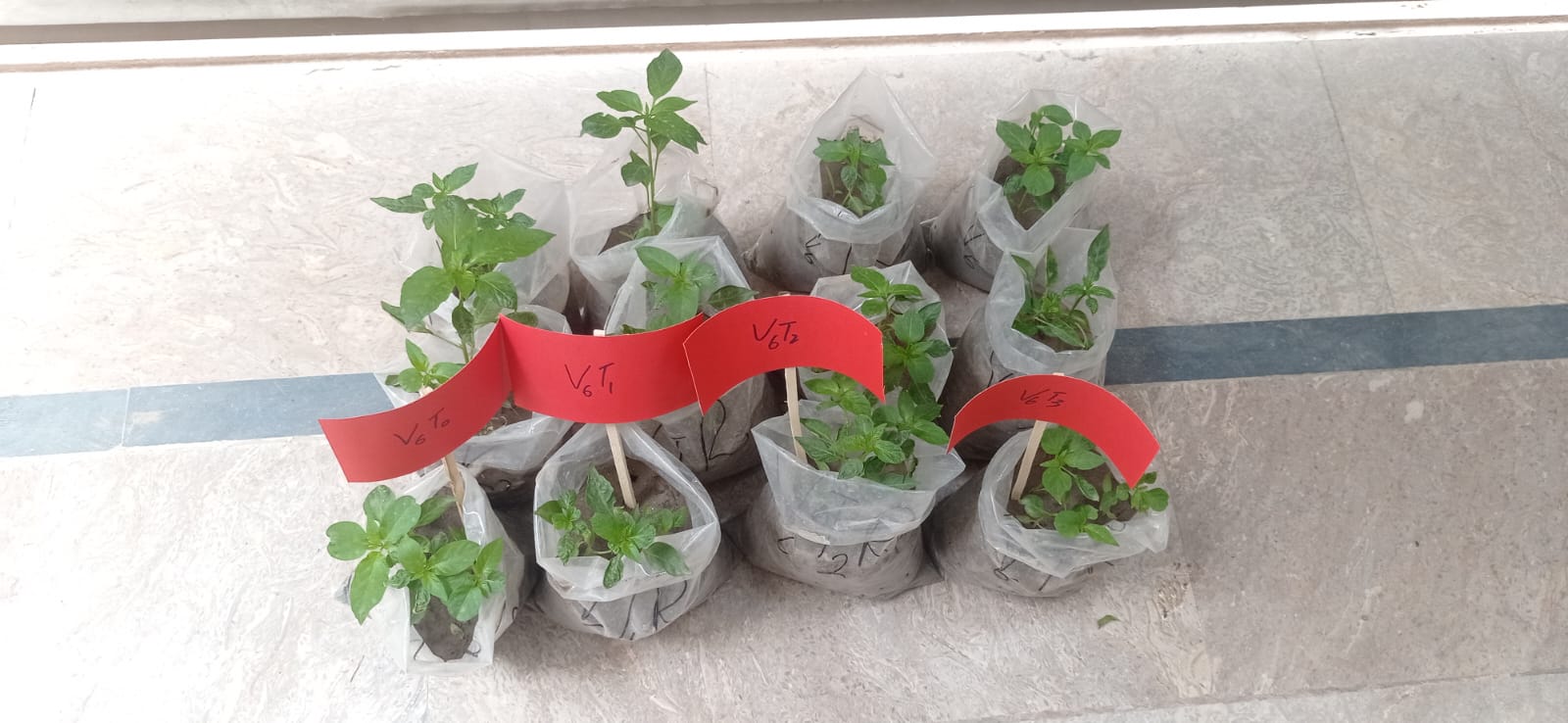


G
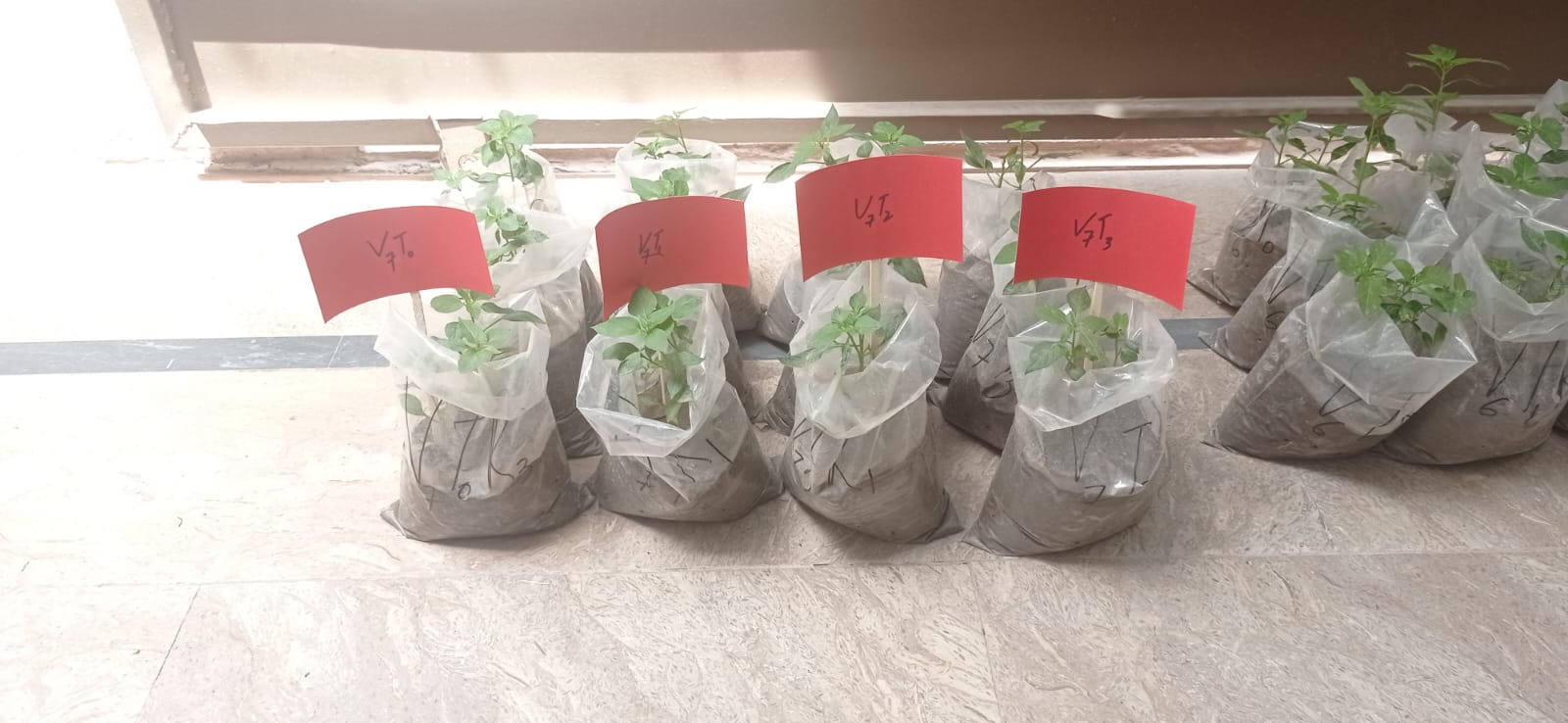


**Figure S1:** Cultivated chili varieties (i.e., V1 (Hybrid) = A, V2 (Desi) = B, V3 (Sathra) = C, V4 (G-916) = D, V5 (BR-763) = E, V6 (BG-912) = F and V7 (F1-9226) = G) grown under cadmium stress.

A
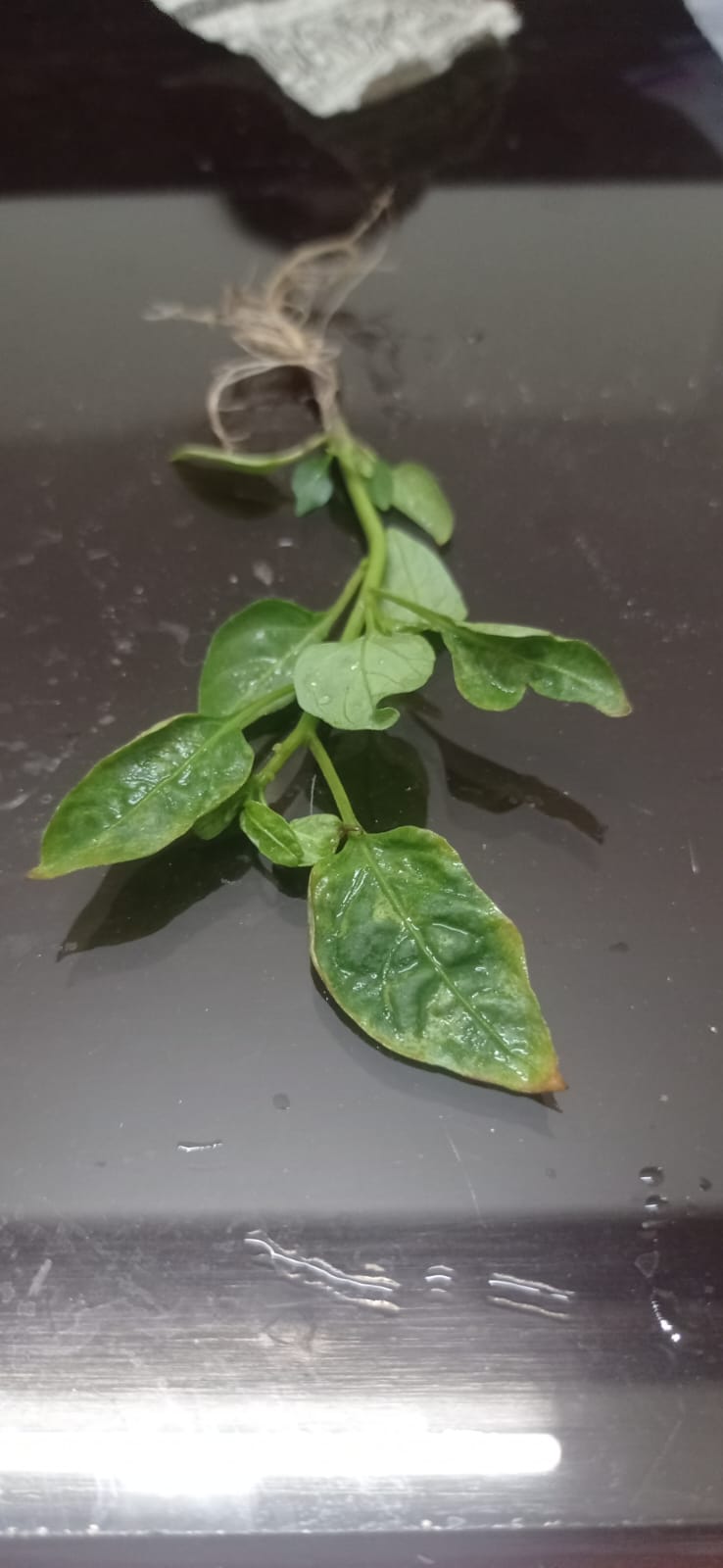


B
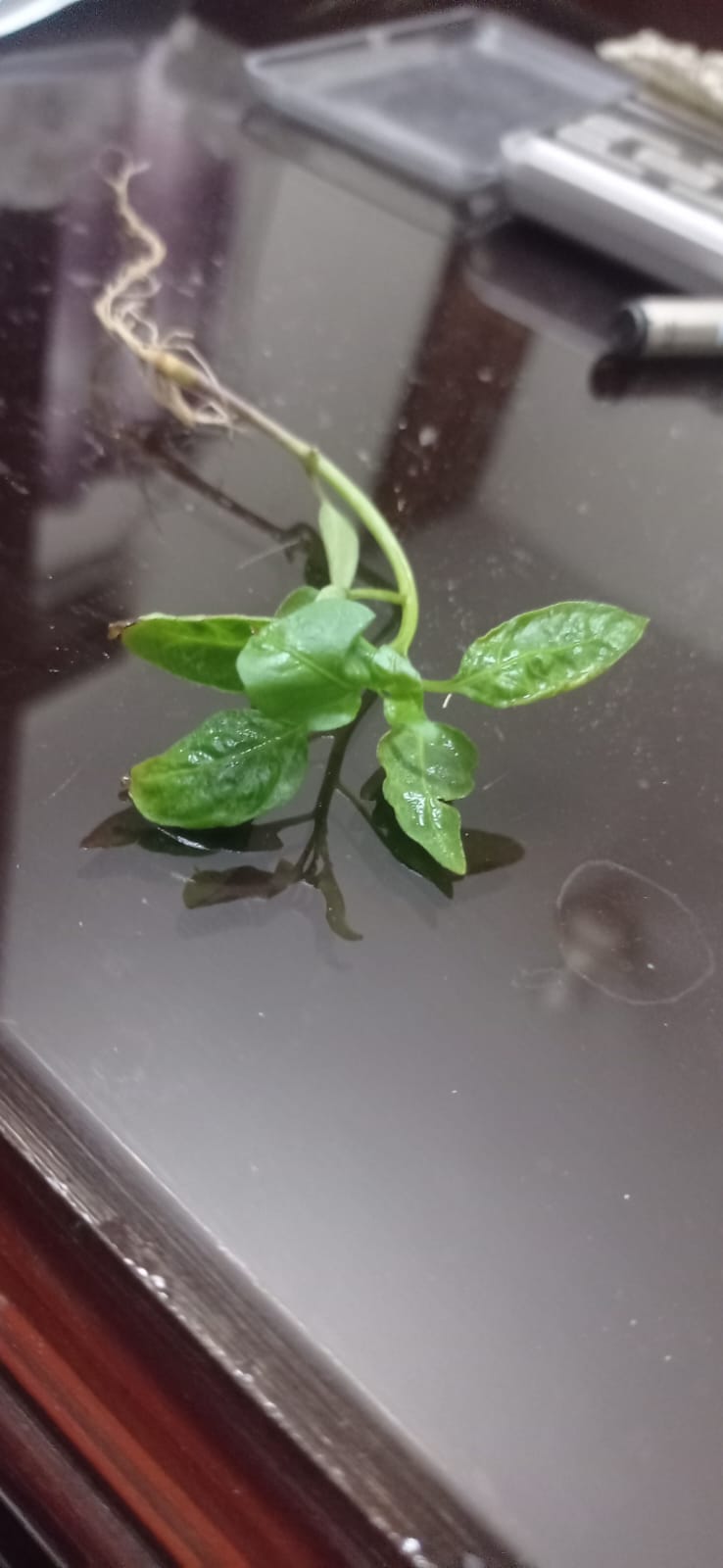


**Figure S2:** Comparison of control (A) and Cd stress (B) plants
